# A second component of the SltA-dependent cation tolerance pathway in *Aspergillus nidulans*

**DOI:** 10.1016/j.fgb.2015.06.002

**Published:** 2015-09

**Authors:** Laura Mellado, Ana Maria Calcagno-Pizarelli, Robin A. Lockington, Marc S. Cortese, Joan M. Kelly, Herbert N. Arst, Eduardo A. Espeso

**Affiliations:** a Departamento de Biología Celular y Molecular, Centro de Investigaciones Biológicas, CSIC, Ramiro de Maeztu, 9, 28040 Madrid, Spain; b Section of Microbiology, Imperial College London, Flowers Building, Armstrong Road, London SW7 2AZ, UK; c Department of Genetics and Evolution, University of Adelaide, Adelaide, South Australia 5005, Australia; d Dept. of Applied Chemistry, Faculty of Chemistry, University of the Basque Country, Manuel de Lardizabal, 3, 20018 San Sebastian, Spain

**Keywords:** Cation-stress response, pH regulation, Signaling pathway, Mutation, Vacuolar protein sorting, Suppressor

## Abstract

The transcriptional response to alkali metal cation stress is mediated by the zinc finger transcription factor SltA in *Aspergillus nidulans* and probably in other fungi of the pezizomycotina subphylum. A second component of this pathway has been identified and characterized. SltB is a 1272 amino acid protein with at least two putative functional domains, a pseudo-kinase and a serine-endoprotease, involved in signaling to the transcription factor SltA. Absence of SltB activity results in nearly identical phenotypes to those observed for a null *sltA* mutant. Hypersensitivity to a variety of monovalent and divalent cations, and to medium alkalinization are among the phenotypes exhibited by a null *sltB* mutant. Calcium homeostasis is an exception and this cation improves growth of *sltΔ* mutants. Moreover, loss of kinase HalA in conjunction with loss-of-function *sltA* or *sltB* mutations leads to pronounced calcium auxotrophy. *sltA sltB* double null mutants display a cation stress sensitive phenotype indistinguishable from that of single *slt* mutants showing the close functional relationship between these two proteins. This functional relationship is reinforced by the fact that numerous mutations in both *slt* loci can be isolated as suppressors of poor colonial growth resulting from certain null *vps* (*vacuolar protein sorting*) mutations. In addition to allowing identification of *sltB*, our *sltB* missense mutations enabled prediction of functional regions in the SltB protein. Although the relationship between the Slt and Vps pathways remains enigmatic, absence of SltB, like that of SltA, leads to vacuolar hypertrophy. Importantly, the phenotypes of selected *sltA* and *sltB* mutations demonstrate that suppression of null *vps* mutations is not dependent on the inability to tolerate cation stress. Thus a specific role for both SltA and SltB in the VPS pathway seems likely. Finally, it is noteworthy that SltA and SltB have a similar, limited phylogenetic distribution, being restricted to the pezizomycotina subphylum. The relevance of the Slt regulatory pathway to cell structure, intracellular trafficking and cation homeostasis and its restricted phylogenetic distribution makes this pathway of general interest for future investigation and as a source of targets for antifungal drugs.

## Highlights

• SltB is a novel component of the cation stress responsive pathway.
• Loss of SltB function results in sensitivity to elevated extracellular concentrations of cations and to alkalinity.
• SltB is involved in signaling to transcription factor SltA.
• SltA regulates expression of *sltB*.
• The Slt pathway is unique to fungi from the pezizomycotina subphylum.

## 1 Introduction

Survival and growth of microorganisms in adverse environments and the ability to benefit maximally from any environment depend upon efficient sensing of and consequent adaptation to the environment. Fungi have developed genetic-based strategies to tolerate a wide range of environmental stresses such as variations in pH, temperature, nutrient availability, reactive oxygen species, osmolarity and ionic strength. The ambient pH response in fungi has been thoroughly studied in many species because of its general importance for viability, adaptation and pathogenicity. For example, secreted proteins (e.g., phosphatases and proteases) and metabolites (e.g., toxins and antibiotics) are useful only if produced at pHs reasonably near their maximal activity (Peñalva and Arst, 2002, 2004). Nutrient availability requires a plasma membrane transport system capable of functioning at ambient pH as well as the presence of the nutrient. Sudden ambient pH changes induce stress on many cellular functions such as nutrient availability, protein function and membrane potential. In addition ambient pH influences fungal virulence in both animals and plants and the major genetic regulatory system for adaptation to ambient pH is crucial to pathogenicity, e.g., (Bertuzzi et al., 2014; Caracuel et al., 2003).

In the model filamentous ascomycete *Aspergillus nidulans*, tolerance of alkaline ambient pH requires the activities of, at least, three zinc-finger transcription factors: PacC, CrzA and SltA (Spielvogel et al., 2008; Tilburn et al., 1995). PacC (Rim101p in *Saccharomyces cerevisiae*) mediates changes in gene expression in response to neutral or alkaline pH (Espeso and Arst, 2000; Espeso and Peñalva, 1996; Tilburn et al., 1995). Activation of PacC requires two proteolytic steps, converting the full length PacC72 to the truncated forms PacC53 and PacC27, respectively, with the latter required for the transcriptional response to alkaline pH (Diez et al., 2002). Only the first proteolytic step is pH-dependent and the ambient pH signal is transduced through the Pal pathway, comprised of six dedicated gene products, to mediate proteolysis of the primary form of the 72 kDa form of PacC to the processing intermediate 53 kDa form (reviewed in Peñalva et al. (2008)). PalB, a calpain family cysteine protease, is almost certainly responsible for PacC72 cleavage. PalA, a Bro1 domain protein, interacts with PacC72 and recruits Vps32 together with the PalB protease. PalH is a transmembrane protein which likely perceives the alkaline pH signal and PalH membrane localization is assisted by PalI, another transmembrane protein. PalC, another Bro1 domain protein, and PalF, an arrestin-like protein, and a number of components of the endosomal sorting complexes required for transport (ESCRTs) are also essential to the function of the plasma membrane-based pH signaling and proteolysis complex (reviewed in Peñalva et al. (2014)).

CrzA (Crz1p in *S*. *cerevisiae*) is a phospho-protein. Different phosphorylation states have been identified in response to elevated calcium levels or alkaline pH (Hernández-Ortiz and Espeso, 2013). GskA, a homologue of human Gsk3α/β kinases, and casein kinase I, CkiA (Apostolaki et al., 2012), phosphorylate CrzA. Calcineurin phosphatase dephosphorylates CrzA (Hernández-Ortiz and Espeso, 2013). The presence of divalent cations such as calcium or manganese or medium alkalinization induces nuclear accumulation of CrzA and calcineurin phosphorylation of CrzA is responsible for its nuclear transport (Hernández-Ortiz and Espeso, 2013).

O’Neil and collaborators characterized the gene *sltA* (*stzA*) in *A*. *nidulans*, which encodes a transcription factor with three zinc fingers of the C_2_H_2_ class (O’Neil et al., 2002). *sltA1* mutant strains, lacking the C-terminal region after the zinc finger domain, are sensitive to a variety of conditions, such as elevated levels of sodium chloride or arginine, UV light and the alkylating mutagen N-methyl-N′-nitrosoguanidine (MNNG) (Clement et al., 1996). *sltA* deletion strains display sensitivity to high concentrations of a number of monovalent cations including Li^+^, Na^+^ and K^+^, and the divalent Mg^2+^ cation but not to Ca^2+^. In fact, an elevated extracellular concentration of Ca^2+^ reduces the sensitivity of *sltAΔ* strains to cations and alkalinity (Spielvogel et al., 2008). In contrast to PacC and CrzA homologues which are widely distributed throughout the fungal kingdom, SltA homologues are found only in fungi belonging to the Pezizomycotina subphylum (Chilton et al., 2008; Spielvogel et al., 2008). *Ace1*, a repressor of the cellulase and xylanase genes in *Hypocrea jecorina* (Saloheimo et al., 2000), is the only characterized homologue of SltA. Conservation of the DNA binding domain is high and both SltA and Ace1 recognize the consensus sequence 5′-AGGCA-3′ (Saloheimo et al., 2000; Spielvogel et al., 2008).

Transcriptional roles of SltA are diverse. A negative effect on the expression of *vcxA*, encoding a vacuolar calcium exchanger and a positive effect on the Na^+^ pump ATPase-encoding *enaA* have been described (Spielvogel et al., 2008). Elevated expression of calcium vacuolar ATPase-encoding genes *pmcA* and *pmcB* is seen in a *sltA*^−^ background and is physiologically involved in cytoplasmic Ca^2+^depletion (Findon et al., 2010). Moreover, *sltA*^−^ strains are hyper-vacuolated with an increased number, size and distribution of vacuoles, and calcium remediates this phenotype (Findon et al., 2010). Recently we have proposed a role for *sltA* in morphological development and sterigmatocystin production in *A*. *nidulans* (Shantappa et al., 2013). A possible role for SltA in facilitating intracellular traffic in the absence of endosomal maturation might explain the rescue by loss-of-function mutations in *sltA* of the near-lethal phenotypes caused by a number of *vpsΔ* (vacuolar-protein-sorting deletion) alleles (Calcagno-Pizarelli et al., 2011).

In contrast to knowledge concerning the consequences of SltA absence, little is known about modulation of the activity of this transcription factor. Among the extragenic *vpsΔ* suppressors we isolated are mutations mapping in a locus other than *sltA*. We have designated this other locus *sltB*. Here we identify *sltB* as auto-called gene AN6132. The *sltB* gene can encode a protein of 1272 amino acids with two putative functional domains: a pseudo-kinase domain and trypsin-like protease domain. Similarly to SltA, SltB is restricted to the Pezyzomicotina subphylum. We have constructed and characterized a strain carrying a null allele of *sltB*, shown that its phenotype is indistinguishable from that of a null *sltA* mutant and determined that the two null mutations are non-additive, indicating that *sltB* is an element of the *sltA* stress response pathway. We also show that *sltB* is expressed in a SltA dependent manner.

## 2 Materials and methods

### 2.1 A. nidulans strains, growth conditions and molecular techniques

*A. nidulans* strains used are listed in Table 1. Gene nomenclature follows (Clutterbuck, 1993). Since the *pabaB22* mutation was the only genetic marker uncharacterized by sequence change in the strains used for this work, and to foresee any interference in the *slt* system, we proceeded to its characterization. The *p*-aminobenzoate auxotrophic mutation *pabaB22* maps in chromosome IV. Thus, we searched in this chromosome for putative homologues of genes belonging to the *p*-aminobenzoate and folic acid biosynthetic pathway from *S*. *cerevisiae*. We identified AN10991 as a putative homologue of *ABZ2* gene of *S*. *cerevisiae*. After sequencing AN10991 coding region we found that *pabaB22* is an A739C transversion (nucleotide numbering relative to translation initiation codon) and consequently Asn247His. The *pabaB22* mutation of strain MAD3682 (Table 1) was complemented in transformation using a wild type version of the AN10991 coding region. Thus, AN10991 was designated as the *pabaB* gene, putatively encoding 4-amino-4-deoxychorismate lyase. This mutation does not affect the *slt* system (data not shown). Strains were cultured in standard *Aspergillus* minimal (AMM) and complete (ACM) media (Cove, 1966) at 37 °C. For the isolation of SltB cDNA, total RNA was extracted from mycelia of MAD2733 grown in liquid AMM containing 1% d-glucose and 5 mM ammonium tartrate as main carbon and nitrogen sources, respectively, for 16 h at 37 °C. *sltB* cDNA was synthesized using the first strand synthesis kit from Roche, using oligo dT for producing the first strand and this was used in PCR using specific oligonucleotides in the 5′UTR and 3′UTR. Growth tests were carried out in AMM supplemented, where indicated, with 1 M or 0.6 M KCl, 1 M NaCl, 0.3 M LiCl, 0.2 M or 0.05 M MgCl_2_, 0.01 M CaCl_2_, 0.1 M Na_2_HPO_4_ or 25 mM TrisHCl to adjust medium pH to 8. Colony growth was scored at 48 and 72 h. Strains were generated by crossing or transformation. *A*. *nidulans* transformation was performed as described previously in Tilburn et al. (1983). Positive transformants were selected on the basis of auxotrophies complemented by heterolous orthologues of *pyrG89* or *riboB2* (e.g., complementation of *pyrG89* by the *pyr*-4 gene from *N*. *crassa* in pRG3-AMA1 plasmids in media lacking pyrimidines), or for mutant *sltB* strains based on reversal of sensitivity to elevated concentrations of potassium (0.6 M KCl) in the media. Other selection procedures are specifically described in the text (see also next section). Construction of the genomic DNA library in the pRG3-AMA1 plasmid and its use in the identification of genes by complementation is described in Osherov and May (2000).

Genomic DNA and total RNA extraction for northern and Southern blotting were carried out following standard protocols as described in Etxebeste et al. (2008). For Southern blotting specific probes were generated for each construct to provide an adequate pattern of bands in combination with the restriction enzyme selected, usually a combination of 5′ and 3′UTR labeled regions for the generation of null alleles in *sltA*, *sltB* and *pskA*.

### 2.2 Deletion cassettes and suppressors

The *sltB*, *sltA* and *pskA* deletion cassettes were constructed as gene-replacement cassettes containing the *riboB* or *pyrG* gene of *Aspergillus fumigatus* as the selection marker by fusion PCR (Markina-Iñarrairaegui et al., 2011). Primers used are listed in Table S1, named as PP1/PP2 (promoter primers 1 and 2), SMP1/GFP2 (selectable marker 1/green fluorescence primer 2) and GSP3/GSP4 (gene specific primers 3 and 4). Pyrimidine or riboflavin prototrophic transformants were analyzed by Southern blotting. Spontaneous suppressor mutations in *vpsΔ* strains were obtained from transformation plates following the procedure described in Calcagno-Pizarelli et al. (2011), and *sltA* and *sltB* mutations were identified by sequencing.

### 2.3 Gene expression analyses

Wild-type and mutant strains were cultivated in supplemented liquid AMM for 16 h at 37 °C, after which the culture medium was made 1 M with respect to NaCl concentration or 10 mM with respect to CaCl_2_ concentration where indicated. Mycelia were incubated for one hour and samples were collected at time 0 (absence of added NaCl or CaCl_2_), 15, 30 and 60 min (presence of NaCl or CaCl_2_ where indicated). Total RNA extraction and northern protocols followed (Garzia et al., 2009). Transcripts of *sltA* and *sltB* were detected using specific genomic probes amplified by PCR (oligonucleotides listed in Table S1). A 1573 bp probe (covering 56% of the ORF) was used for *sltA* and a 1452 bp probe (covering 35% of the ORF) was used for *sltB*. Transcript levels of the housekeeping glyceraldehyde-3-phosphate dehydrogenase gene (*gpdA*) was used as internal control for normalization. A PhosphorImager imaging plate, BAS-IP-MS 2040, was used to detect radioactive mRNA bound probes with development using a FLA-5100 Reader (FujiFilm). Quantification of band intensities was performed using Multi-Gauge V3.0 software (Fujifilm).

### 2.4 Microscopy

Conidiospores of *A*. *nidulans* strains were inoculated in watch minimal medium (WMM, Peñalva, 2005) with 17.5 mM NaH_2_PO_4_, 7.5 mM NaHPO_4_, 10 mM ammonium (+)-tartrate and 5 mM glucose and grown for 16–18 h at 25 °C. Staining of vacuoles with CMAC blue (10 μM final concentration) and external and internal membranes with FM4-64 (5 μM final concentration) was as described in Calcagno-Pizarelli et al. (2011).

### 2.5 Phylogenetic analysis

Multiple sequence alignments were made using the ClustalX program (Thompson et al., 1997). Alignments visualization/analysis were performed with Genedoc and Jalview programs. Hidden Markov Models, HMM, were generated through the Institut Pasteur Mobyle server (http://mobyle.pasteur.fr/). Phylogenetic trees were generated using Mega 5.2 software (Tamura et al., 2011), following the Neighbor-Joining method with 100,000 replicates per node. The Peptidase Database, MEROPS (http://merops.sanger.ac.uk/) and the clustalw2 application in EBI (http://www.ebi.ac.uk/Tools/msa/clustalw2/) were used for sequence similarity searching and analysis of the SltB protease domain.

## 3 Results

### 3.1 Isolation of a null vps suppressor identifies a new slt locus, sltB

**Fig. 1:**
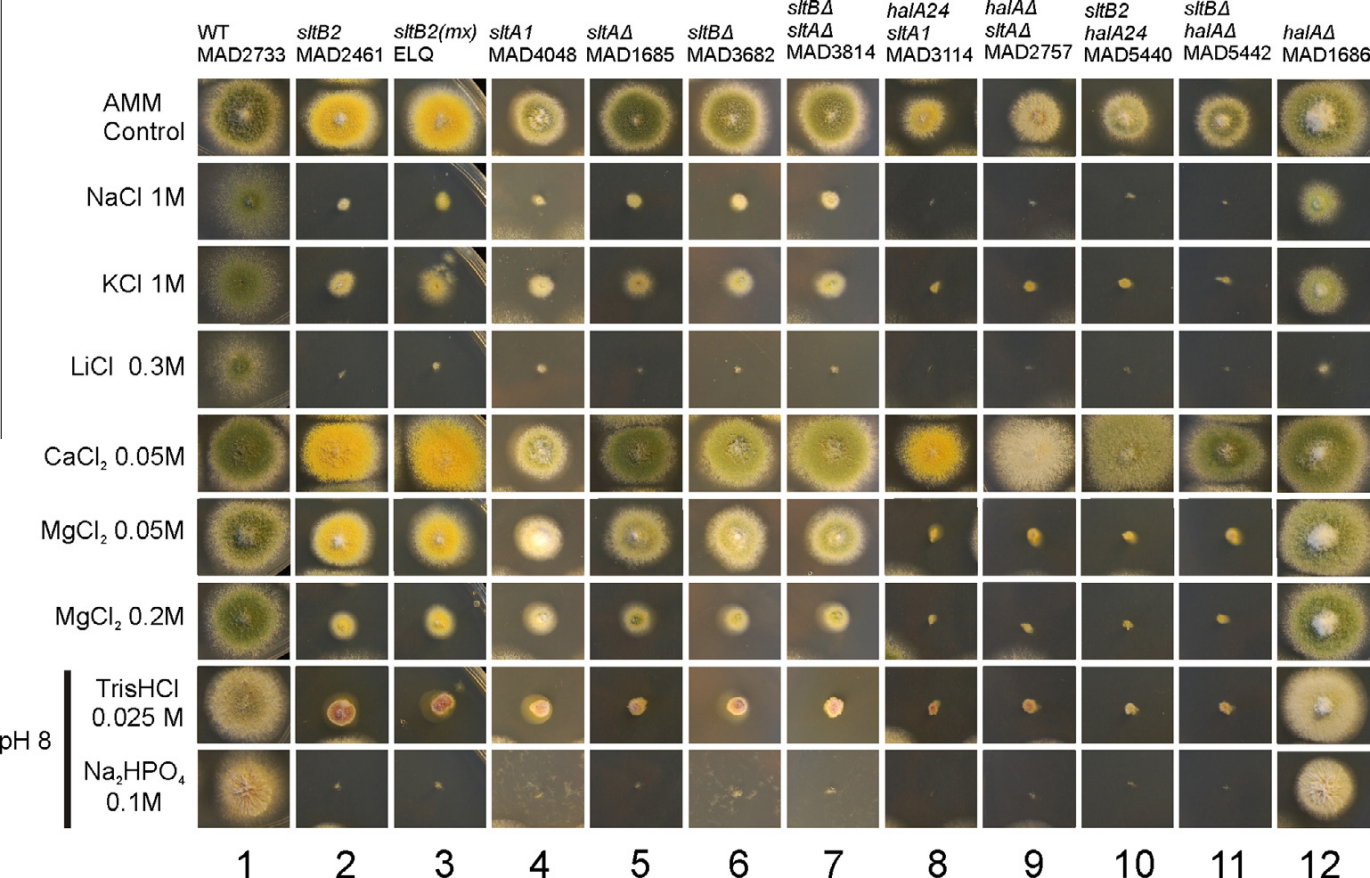
Phenotypes of *sltB mutants*. Phenotypic analysis of strains carrying *slt*^−^ mutations in media supplemented with elevated concentrations of various cations or adjusted to pH 8 with Na_2_HPO_4_ or Tris HCl. Column 1 shows growth of wild type strain (WT) MAD2733. The cation and alkalinity sensitivities of mutant *sltB2* and *sltBΔ* strains are comparable to those of *sltA*^−^ mutants (columns 2–6). *sltB sltA* double null mutants are indistinguishable from single *slt* null mutants (compare columns 5–7). The presence of the *halA24* or *halA*Δ alleles results in even more extreme hypersensitivity to all stress conditions tested with the exceptions alkalinity and calcium concentration elevation. The reduced growth of *slt hal* double mutant strains (columns 8–11) on AMM and their improved growth in the presence of 50 mM calcium is indicative of the calcium auxotrophy (Findon et al., 2010). *halA*Δ strains are hypersensitive to lithium and have reduced growth on medium containing 1 M Na^+^ or 1 M K^+^ but have wild type responses to calcium, magnesium and alkaline pH (column 12).

The first evidence of the existence of an additional gene in the SltA-dependent cation tolerance pathway came from a mutation, initially named mx, which arose spontaneously and suppresses the very poor growth of a *broA^vps31^Δ* strain. This mutation, now designated *sltB2* arose at some point in the course of construction, growth or haploidization of a diploid heterozygous for *broA^vps31^Δ*. BroA is the homologue of *S*. *cerevisiae* Bro1p [Vps31, a cytoplasmic class E vacuolar protein sorting (VPS) factor (R.A.L. and J.M.K., unpublished)]. Strains carrying *sltB2* resemble null *sltA* mutants in being hypersensitive to elevated concentrations of monovalent cations potassium, sodium and lithium, divalent magnesium ion and alkaline media (Fig. 1, compare columns 1–5) (Calcagno-Pizarelli et al., 2011). Genetic analyses showed the absence of linkage of *sltB2* to *sltA* and this mutation was located to chromosome I using the parasexual cycle (data not shown). Preliminary meiotic mapping suggested linkage of *sltB* to *suAadE20* on the left arm of chromosome I.

### 3.2 Identification of the sltB locus

As *sltB2* is recessive, we used the genomic library based on the auto-replicative pRG3-AMA1 plasmid (Osherov and May, 2000) to reverse the sensitive phenotype of the *sltB2* strain ELQ (Table 1) to 50 mM sodium acetate in protoplast regeneration plates (containing 1 M sucrose as osmotic-stabilizing agent). Total DNA, genomic plus recombinant pRG3-AMA1 plasmids, was extracted from 50 mM sodium acetate tolerant transformants and used to transform *Escherichia coli*. Two plasmids, designated p4.6 and p6.2, were recovered. To verify that these plasmids complement *sltB2*, they were tested separately for *sltB2* complementation in the ELQ strain for sensitivity to 0.6 M KCl and in a *sltB2*; *pyrG89* strain (MAD5440, Table 1). In the latter, we first selected for rescue of the *pyrG89* pyrimidine auxotrophy by the orthologous *Neurospora crassa pyr*-4 gene present in the pRG3-AMA1 plasmid and then tested for tolerance to 1 M NaCl, 0.6 M KCl and alkalinity. Both plasmids are able to complement *sltB2* individually.

**Fig. 2:**
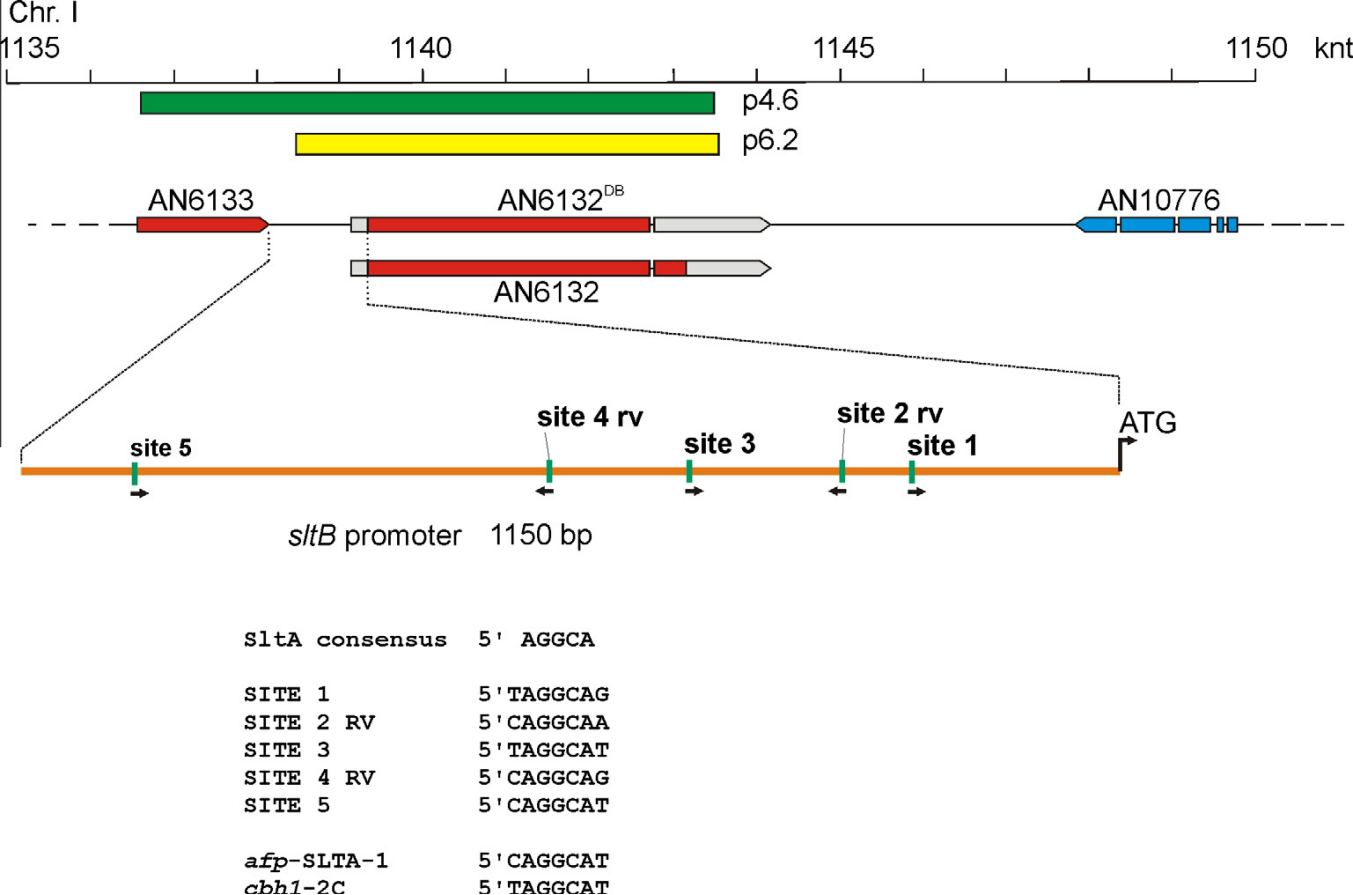
Schematic representation of the AN6132 locus. Based on the database, a diagram of the *sltB* locus, AN6132, and adjacent loci is shown. At the top is a scale of the region between 1135 and 1150 knt of chromosome I. The green and yellow bars represent the inserts in plasmids p4.6 and p6.2, respectively, as they relate to the relevant region of chromosome I. The current database version of *sltB* is indicated with the name AN6132^DB^, and below it is the version proposed in this work. A magnification of the AN6133-*sltB* intergenic region including the number, distribution and sequences of putative SltA binding sites is shown. The sequences of the SLTA-1 site from the *Aspergillus giganteus afp* gene and site 2C from the *Trichoderma reesei cbh1* gene are shown for reference (Spielvogel et al., 2008). (For interpretation of the references to color in this figure legend, the reader is referred to the web version of this article.)

To identify the genomic region present in p4.6 and p6.2 we sequenced flanking regions of inserts, using oligonucleotides PRGUP and PRGDW (Table S1), and searched the *A*. *nidulans* genome data base (www.aspgd.org). Plasmid p4.6 contains a 7958 bp genomic insert which covers a region between coordinates 1,136,528 and 1,143,486 of chromosome I. Plasmid 6.2 carries a fragment of 5031 bp and covers coordinates 1,138,516–1,143,547 of chromosome I (Fig. 2). Inserts of the two plasmids overlap in the genomic region containing locus AN6132, heretofore a hypothetical gene with an undefined function.

### 3.3 Absence of SltB is phenotypically identical to absence of SltA

Initial attempts to identify the *sltB2* mutation by PCR amplification of AN6132 locus using specific primers were unsuccessful. Southern blot analyses showed that the *sltB2* mutant lacked the entire predicted AN6132 coding sequence in a deletion of at least 9 kb (data not shown). As this deletion might affect flanking genes, strains null only for *sltB* were generated following standard procedures (Markina-Iñarrairaegui et al., 2011). Null *sltB* transformants were obtained by precisely deleting the coding sequence and replacing it with the *pyrG* gene from *A*. *fumigatus* (*pyrG^Af^*) as selectable marker for pyrimidine protrophy. Strain MAD1427 (Table 1) was used as a recipient for transformation. Null *sltB* transformants grew poorly in media containing elevated concentrations of diverse cations or when the pH was adjusted to 8 (Fig. 1, column 6). These sensitive phenotypes of a *sltBΔ* strain (MAD3624) were indistinguishable from those shown by the *sltB2* strain, and, more interestingly, from those of a null *sltA* strain (as briefly noted in Calcagno-Pizarelli et al. (2011)) (Fig. 1, compare columns 3, 5 and 6).

To characterize further the deletion phenotypes, we constructed a *sltB sltA* double deletant strain. Firstly we generated a new null *sltB* mutant by replacing the *sltB* coding sequence with the *riboB* gene from *A*. *fumigatus* (*riboB^Af^*) and using strain MAD1427 as recipient. Riboflavin-prototrophic transformants were obtained and have identical phenotypes to those of the *sltBΔ::pyrG^Af^* null mutants. Using one of these *sltBΔ::riboB^Af^* transformants (MAD3669), we generated by transformation a null *sltA* allele by replacing the *sltA* coding region with *pyrG^Af^*, as above. Several *sltBΔ::riboB^Af^ sltAΔ::pyrG^Af^* strains were obtained. Double mutant strain MAD3814 showed hypersensitive phenotypes to elevated concentrations of monovalent cations (Na^+^, K^+^, Li^+^), the divalent magnesium cation, and alkalinity, identical with those displayed by single null *sltA* and *sltB* strains (Fig. 1, column 7). Addition of calcium to the medium improved growth of all *slt*^−^ strains (Fig. 1). Thus null mutations in *sltA* and *sltB* are non-additive (as briefly noted in Calcagno-Pizarelli et al. (2011)).

Double mutants lacking SltA and the kinase HalA in *A*. *nidulans* have a pronounced calcium auxotrophy (Findon et al., 2010). Fig. 1 shows the growth-stimulating effect of adding 50 mM calcium chloride to *halAΔ sltAΔ* (MAD2757) and *halA24 sltA1* (MAD3114) double mutants. Double mutants *sltB2 halA24* (MAD5440) and *sltBΔ halAΔ* (MAD5442) strain also required calcium supplementation for normal growth and conidiation (pRG3-AMA1 plasmids p4.6 and p6.2 complement this deficiency in strain *sltB2 halA24*, MAD5440, data not shown). In contrast to calcium, magnesium inhibits growth of null *sltA* and *sltB* strains (Fig. 1). Double *halA*^−^ *slt*^−^ mutants are extremely sensitive to Mg^2+^. Fig. 1 shows the extreme sensitivity to 50 mM MgCl_2_ of various *sltB*^−^ *halA*^−^ double mutants as compared to the single mutants. Loss of HalA kinase function alone does not affect sensitivity to magnesium (Fig 1).

**Fig. 3:**
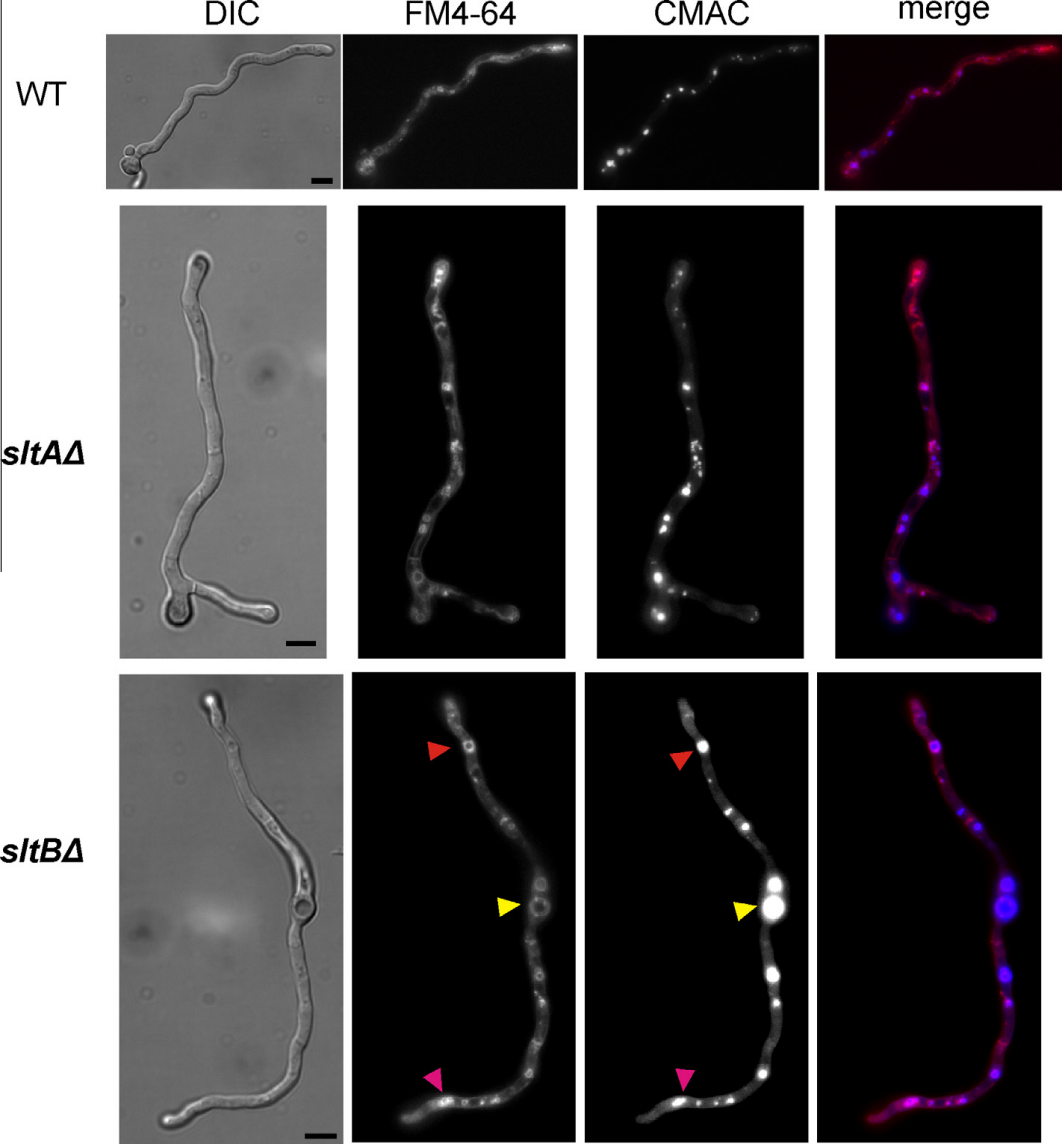
Vacuole distribution in null *sltB* cells. Absence of SltB is equivalent to absence of SltA with regard to the vacuolar system. Vacuoles, visualized using CMAC (vacuole compartment) and FM4-64 (vacuole membrane), have abnormally large volumes and are distributed in part abnormally close to the hyphal tips in null *sltA* (see also Calcagno-Pizarelli et al. (2011)) and null *sltB* cells. Bars = 5 μm.

*sltA*^−^ mutations increase the number and size of vacuoles and extend their distribution much closer to germling tips (Calcagno-Pizarelli et al., 2011; Findon et al., 2010). Fig. 3 shows that *sltBΔ* results in hypertrophy of the vacuolar system very similar to that seen in *sltAΔ* strains. In Fig. 3, the yellow arrowhead adjacent to the *sltBΔ* germling indicates a large vacuole in the conidiospore whereas the red arrowhead indicates a large vacuole near the tip. At the opposite tip, the magenta arrowhead indicates a concentration of vacuoles. All of these phenotypic similarities indicate a direct genetic and possibly molecular involvement between SltB and SltA.

### 3.4 Gene structure and domain analysis of SltB

**Supplementary Fig. S1:**
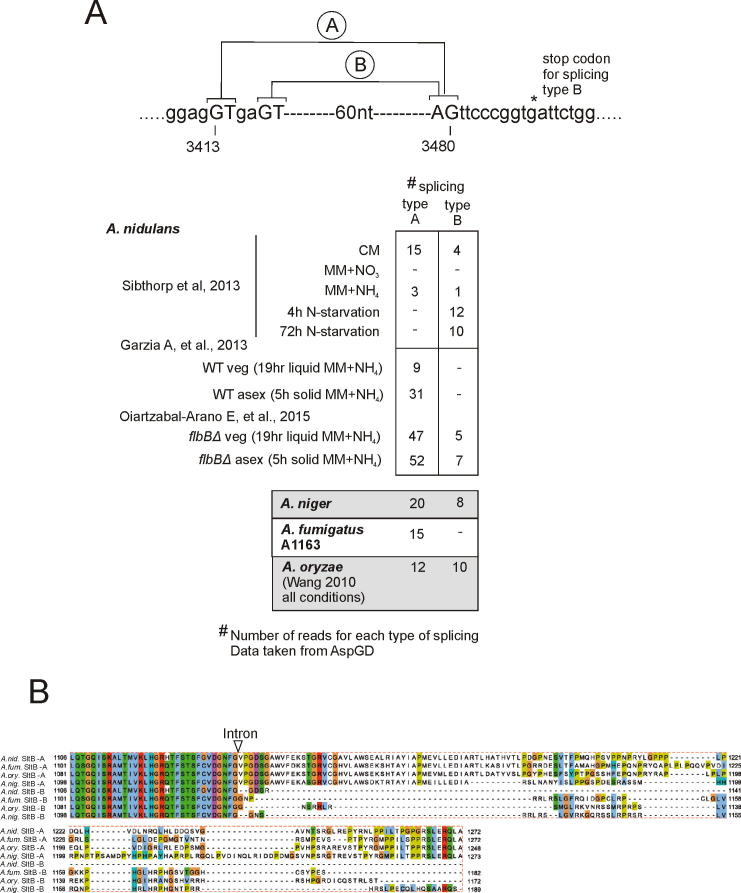
The alternative processing of *sltB* mRNA. (A) Alternative splicing of the unique intron in the *sltB* coding sequence based on the data from available RNA-seq. The acceptor site at position 3480-1 remains constant. Splicing type A uses donor site at position 3413-4 and splicing type B uses the donor site at position 3417-8. This alternative splicing is observed in RNA-seq data from other *Aspergillus* species, i.e. *A. niger*, *A. fumigatus* and *A. oryzae* (source AspGD, http://www.aspgd.org). The number of reads (#) covering each type of splicing is shown. For *A. nidulans* the number of reads from RNA-seq experiments performed under different growth conditions is shown (see Garzia et al. (2013) and Oiartzabal-Arano et al. (2015)). (B) Multiple alignment of the amino acid sequences of *A. nidulans* SltB and its homologues in *A. niger*, *A. fumigatus* and *A. oryzae*, derived from each type of splicing. The alignment of the encoded sequence from the region of splicing to the stop codon is shown. Note the conservation of sequences when using the type A splicing.

In the course of this work, the open reading frame delineation for AN6132 was modified in the *Aspergillus* database due to inclusion of RNAseq data (Sibthorp et al., 2013). The difference with the previous prediction is the location of the donor splice site of the single intron present in the coding sequence. However, a detailed analysis of RNA-seq data from *A*. *nidulans* and other *Aspergilli* (*A*. *niger*, *A*. *fumigatus* and *A*. *oryzae*) indicated the existence of an alternative splicing of the intron, detected in all species (see Fig. S1). In *A*. *nidulans*, the donor splice site varies depending on the amount and type of nitrogen source used by Sibthorp and collaborators (Sibthorp et al., 2013) (Fig. S1). Our previous RNA-seq data using liquid or solid AMM containing ammonium tartrate as the main nitrogen source (Garzia et al., 2013; Oiartzabal-Arano et al., 2015) identified a preferential intron splicing between donor site at nt 3413-4 from the initiator ATG and the invariable acceptor site at nt 3479-80 (see Fig. S1). cDNA sequencing confirmed the limits of the intron sequence as those automatically predicted in previous versions of this locus (i.e. AN6132.2, Figs. 2 and S2) and in our previous RNA-seq experiments using ammonium tartrate as nitrogen source. This splice version of *sltB* and its homologues results in proteins with high conservation along the most C-terminal residues. However, the alternative splicing produced early truncations and poor sequence conservation in frame-shifted sequences (Fig. S1). Consequently, AN6132 encodes a 1272 amino acid polypeptide (Fig. S2).

**Fig. 4:**
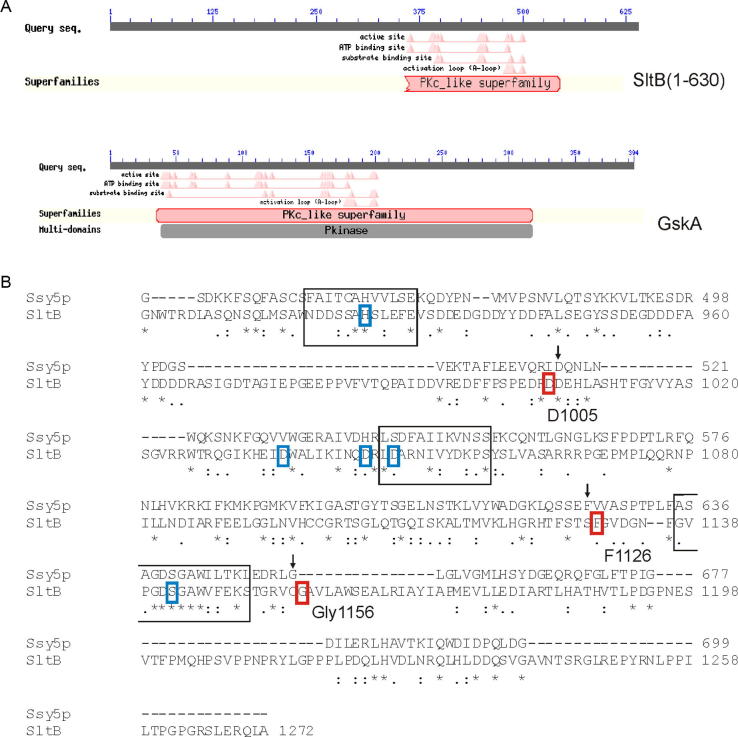
Prediction of functional domains in SltB. (A) Prediction of a protein kinase C (PKc)-like superfamily domain in the N-terminal region of SltB (residues 1-630). For comparison, prediction and distribution of functional residues in the GskA kinase (Hernández-Ortiz and Espeso, 2013) is shown. Diagrams were obtained from the NCBI conserved domains predictor. (B) Alignment between the Ssy5p sequence and the C-terminal region of SltB. The complete Ssy5p sequence, 699 residues, and the SltB region between residues 629 and 1272 were compared using the Blast2p package at NCBI. Black boxes indicate in the alignment those regions proposed by Abdel-Sater et al. (2004) to contain those residues comprising the catalytic triad in Ssy5p. Blue boxes indicate His 925, D1049 and S1142 of SltB, proposed as the catalytic triad in SltB. Because D1049 is a poorly conserved residue among SltB homologues (see Fig. 5), alternative highly conserved Asp residues, D1037 and D1046, are also indicated. Red boxes indicate residues affected by mutations in SltB. (For interpretation of the references to color in this figure legend, the reader is referred to the web version of this article.)

Initial bioinformatics analyses predicted the presence of a putative Ser/Thr kinase domain in the N-terminal moiety of SltB. For instance, the NCBI conserved domain predictor indicated that the region between residues 350 and 550 might be part of a kinase domain (Fig. 4A). However a more detailed analysis showed the absence in SltB of important functional residues for a protein kinase, such as VAIK, HRD and DFG of subdomains II, VIB and VII, respectively, (Hanks and Hunter, 1995). A HMM profile defined using conserved residues between aa 325 and aa 589, identified in fungal genome databases sequences of proteins belonging to the protein kinase family and in addition a number of proteins classified as pseudo-kinases (see also below). The N-terminal moiety of SltB (residues 1–600) might then encompass a pseudo-kinase domain.

Prediction of possible domains in the C-terminal part of SltB (residues 600–1272) was unsuccessful. Searches for possible homologues in fungal genomes allowed us to define conserved residues in this region of SltB. A new HMM domain was identified for the region between residues 1136 and 1194, and searches showed significant similarity to proteins defined as ascomycete specific proteases. Further analyses using MEROPs showed similarity of the SltB 600-1272 moiety to an uncharacterized member of the S64 protease family (PA clan, PF08192) from *Trichophyton tonsurans* (identity 31.3%, e-value 9.10e^−02^). The founding member of this family of fungal serine proteases is the Ssy5 endoprotease of *S*. *cerevisiae*. However the overall sequence conservation between Ssy5 and the C-terminal part of SltB is rather low (25% identity in 68 aa overlap) (Fig. 4B). In fact BlastP searches using the Ssy5 sequence in the *A*. *nidulans* protein database (www.aspgd.org) did not detect a putative homologue. Abdel-Sater and collaborators predicted the putative catalytic triad (His–Asp–Ser) of the serine endoprotease Ssy5 (Abdel-Sater et al., 2004) and a similar search identified these residues in SltB (Fig. 4B). Histidine-939 and Ser-1142 are highly conserved residues among SltB homologues. The alignment of Ssy5 and SltB sequences suggests Asp-1049 as the third residue of a trypsin-like active site, however this is not a highly conserved Asp among homologues (see Fig 6) and Asp-1037 and Asp-1046 might be alternative candidates to complete the catalytic triad in SltB.

### 3.5 Selection of mutations in sltB as suppressors of null vps mutations

**Supplementary Fig. S3:**
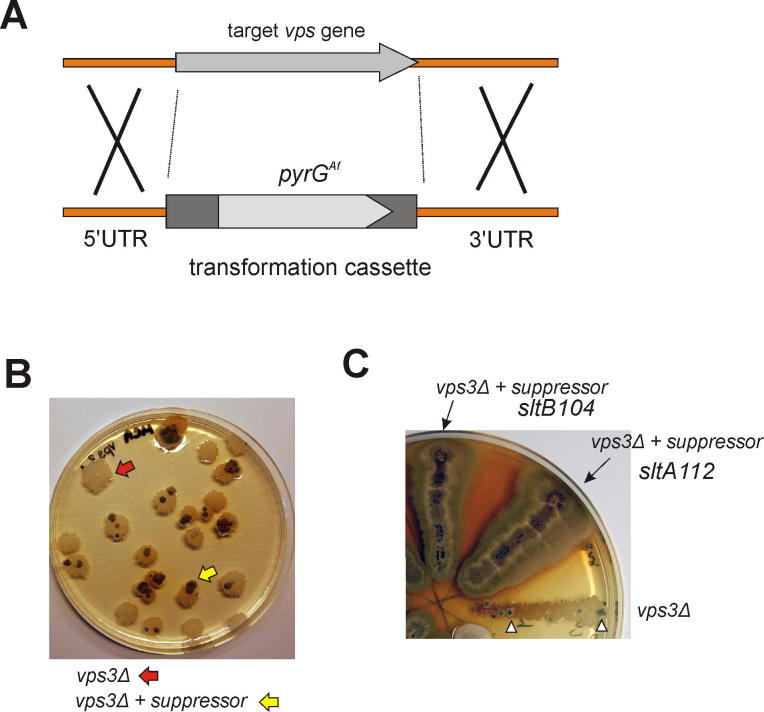
Isolation of loss of function mutations in *slt* genes as suppressors of null *vps* alleles. A strategy to generate null *vps* mutants by gene replacement using as selectable genetic marker the *pyrG* gene from *A. fumigatus* (*pyrG^Af^*). The presence of the 5′ and 3′UTRs in the transformation cassette allows homologous recombination with the target *vps* gene. (B) Sectors (indicated by a yellow arrow) suppressing the strong morphological defect caused by null *vps* alleles (null *vps3* in the image, red arrow) emerge spontaneously on any media (complete medium in the image). (C) Comparison of colonial growth of a null *vps3* strain with other two strains carrying mutation *sltB104* or *sltA112*. The presence of suppressors in the null *vps3* strain is indicated by white arrowheads.

A key feature of *slt*^−^ mutations is their ability to suppress the growth-debilitating effect of certain null *vps* mutations (Calcagno-Pizarelli et al., 2011). This ability enables a facile method to select *slt*^−^ mutations (Fig. S3). Deletion cassettes for these *vps* genes (*vpsΔ::pyrG^Af^*) were transformed into *pyrG89* recipient strains MAD1427, AMC56 and MAD5131 (see Fig. S3A and Calcagno-Pizarelli et al. (2011)). Among homologous integrants of the deletion cassette, sectors showing improved growth appeared readily (see Calcagno-Pizarelli et al. (2011) and Fig. S3B). Sequencing of the *sltA* and *sltB* genes in these suppressor-carrying strains yielded five new mutant alleles for *sltA*, added to those eight mutations previously reported in Calcagno-Pizarelli et al. (2011), and eight mutant alleles for *sltB*, in addition to *sltB2* and *sltBΔ* used to identify this locus. Table 2 displays a list of the new mutations we isolated as suppressors of the *vps20Δ*, *vps32Δ* or *vps3Δ* alleles.

**Fig. 5:**
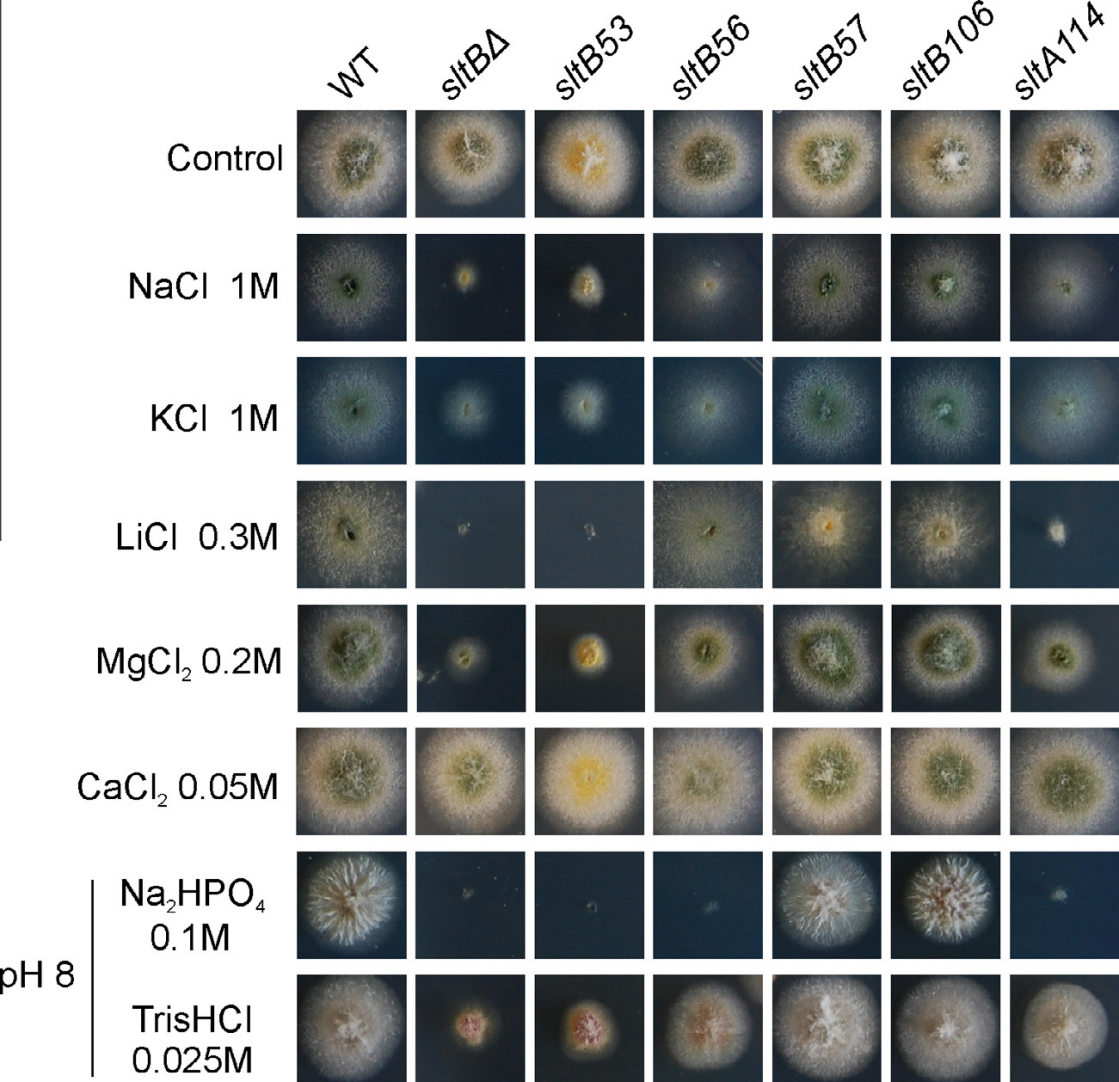
Phenotypic analysis of the *sltB53*, *sltB56*, *sltB57*, *sltB106* and *sltA114* mutations. Mutant alleles *sltB53* (AMC190), *sltB56* (AMC196), *sltB57* (MAD5421), *sltB106* (MAD5425) and *sltA114* (MAD5423) were separated from their original null *vps* backgrounds by crossing. Growth of mutants on AMM to which elevated concentrations of various salts or which was adjusted to pH 8 was compared to a wild-type (MAD2733) and *sltBΔ::pyrG^Af^* (MAD3624) strains. *sltB53* strains behave like *sltBΔ* strains. *sltB56* and *sltA114* have partial loss of function phenotypes. In contrast, *sltB57* and *sltB106* strains behave like wild type strains in all tested conditions.

Interestingly, most *sltA* mutations resulted in truncated versions of the SltA transcription factor, mirroring the results of Calcagno-Pizarelli et al. (2011). The exception is *sltA114* which substitutes Thr for conserved Lys-431 in position six of the α-helix of zinc finger I. Although *sltA114* results in hypersensitivity to 100 mM Na_2_HPO_4_ (pH 8) and lithium cation, only a moderate effect was observed for response to other metal cations (Fig. 5). It is tempting to speculate that Lys431Thr affects a base contact and alters DNA binding specificity (see, as an example, our studies in the transcription factor PacC, Espeso et al. (1997) and references therein).

**Fig. 6:**
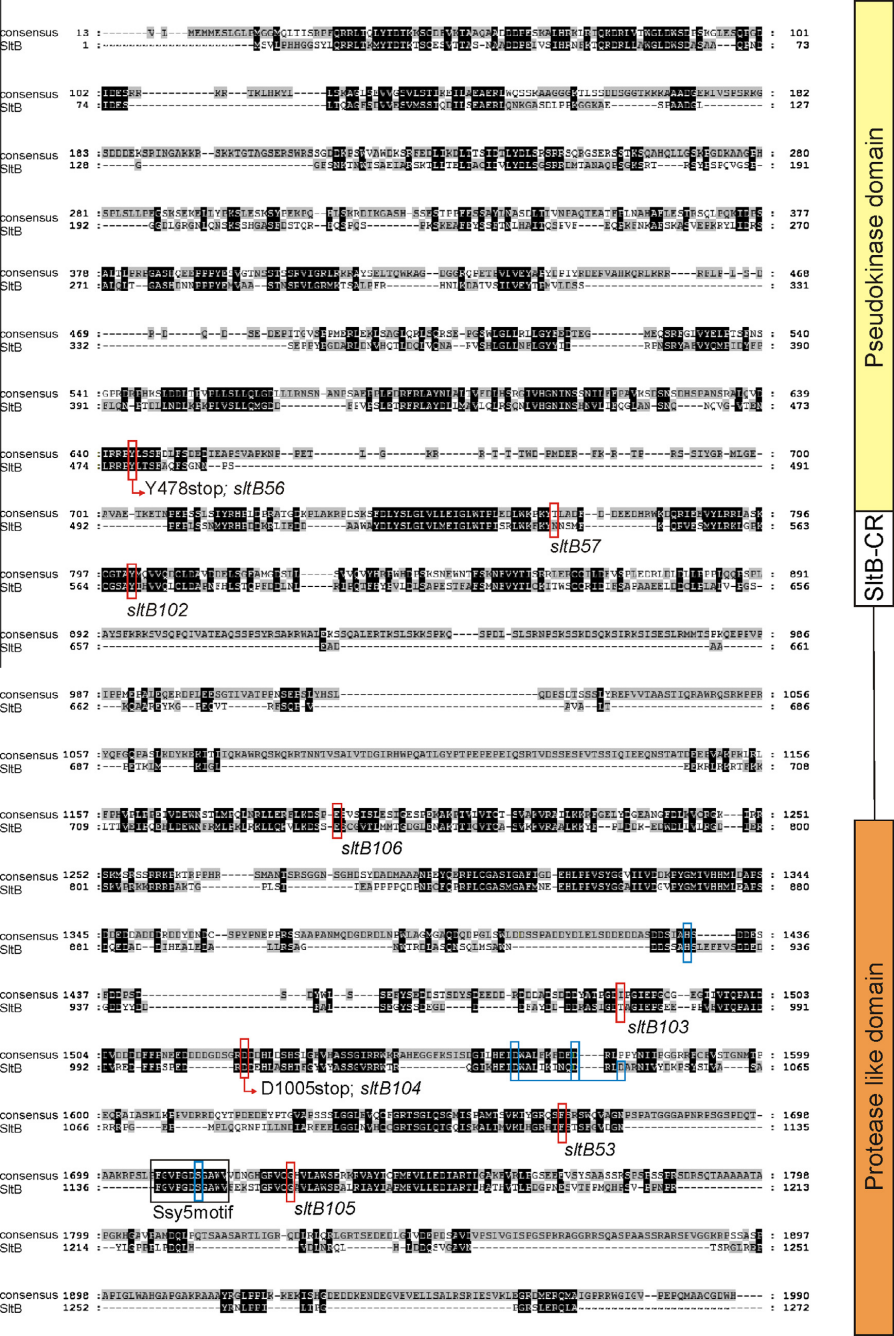
Comparison of SltB with homologues in the Pezizomycotina subphylum. Alignment of the SltB sequence with a consensus sequence generated from a multiple alignment of 69 homologues of SltB present in fungi belonging to the Pezizomycotina subphylum. The positions of extant SltB mutations are indicated. The rectangular box indicates the highly conserved residues also present in the Ssy5p endopeptidase. Blue boxes identify those residues conforming to a putative catalytic triad in the predicted endoprotease domain of SltB (see also Fig. 4). On the right, the diagram indicates possible functional domains in SltB. (For interpretation of the references to color in this figure legend, the reader is referred to the web version of this article.)

In contrast, mutations affecting a single residue by substitution or deletion were more frequent among *sltB* mutations. We have not been able to detect a phenotype for two of these mutations, *sltB57* (ΔN544) and *sltB106* (E743K) beyond their suppression of *vpsΔ* mutations (Fig. 5). *sltB104*, truncating the C-terminal moiety after residue 1004, removes the region of similarity to protease Ssy5 (Figs. 4 and 6). Four other mutations result in changes within the C-terminal moiety: *sltB53* deletes Phe-1126 within the region of similarity to Ssy5 and is phenotypically null (Figs. 4 and 5), *sltB106* substitutes Glu-743 by Lys, *sltB103* (also see below) changes Thr-972 to Pro and *sltB105* substitutes Gly-1156 by Arg, immediately adjacent to the region of Ssy5 similarity (Figs. 4 and 6). The selection and phenotypes of these mutations suggest a functional role for the C-terminal moiety of SltB. Interestingly, *sltB56*, although leading to loss of nearly 2/3 of the protein, does not have a null phenotype (Fig. 5). This might indicate an altered function for a portion of the predicted pseudo-kinase domain when separated from the rest of the protein or, more likely, suggest read through or translational re-initiation. Finally, we note that two mutations might define an additional functional region in SltB: *sltB57* (ΔN544), the *sltB102* (Y568D) affect a conserved region (see below) between the predicted pseudo-kinase and protease domains.

### 3.6 Phylogenetic distribution of sltB genes

**Supplementary Fig. S4:**
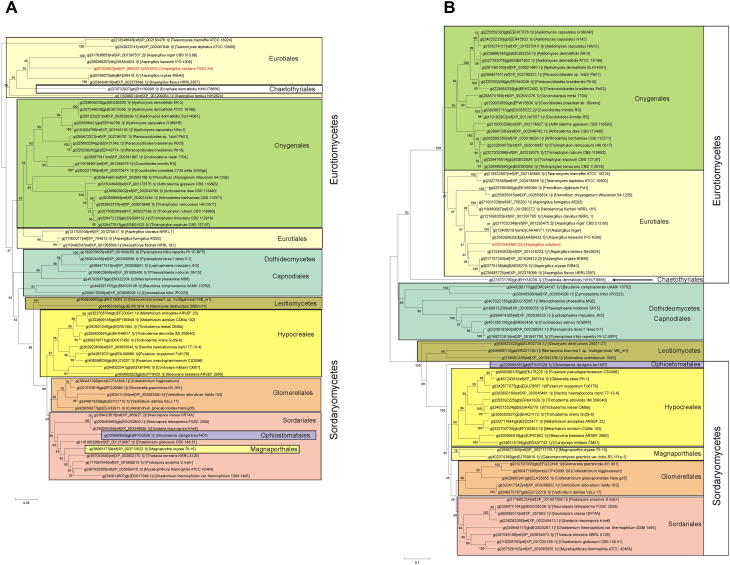
Phylogenetic distribution of SltA and SltB homologues. Blastp searches allowed the identification of putative homologues of SltA (A) and SltB (B) proteins in Pezizomycotina fungi. Multiple alignments of both protein homologues were used to generate these phylogenetic trees using MEGA5 software. Main clades represent taxonomic orders. Sequence conservation of SltA and SltB homologues is high.

In order to identify functional domains as well as possible homologues we performed a range of searches using the available genome databases. SltA homologues are present only in species belonging to the Pezizomycotina fungal subphylum (Chilton et al., 2008; Spielvogel et al., 2008) (Fig. S4A) and this is also the case for SltB (Fig. S4B). All genomes containing a *sltA* homologue also contain a *sltB* homologue and *vice versa*. This suggests that both proteins are required for pathway function in all Pezizomycotina in which they occur.

BlastP searches and more refined searches in Mobyle portal (http://mobyle.pasteur.fr) using HMM profiles generated from highly conserved regions provided 69 sequences of putative homologues. An alignment between the consensus generated from a multiple alignment and the *A*. *nidulans* SltB sequence is shown in Fig. 6. There are numerous blocks of highly conserved amino acids throughout the sequence, corroborating the *sltB* coding sequence. Fig. 6 also shows that most mutations characterized in *sltB* affect highly conserved residues among SltB homologues. Also conserved is a short motif identified in the alignment of SltB with the Ssy5p protease (designated as Ssy5 motif, Fig. 6). Taken together, the distribution of *sltB* mutations, multiple alignment and domain searches suggested that SltB can be divided into three regions: an N-terminal pseudo-kinase domain, followed by a highly conserved region, designated SltB-CR, a non-conserved region probably acting as linker and the protease-like domain (Fig. 6).

**Supplementary Fig. S5:**
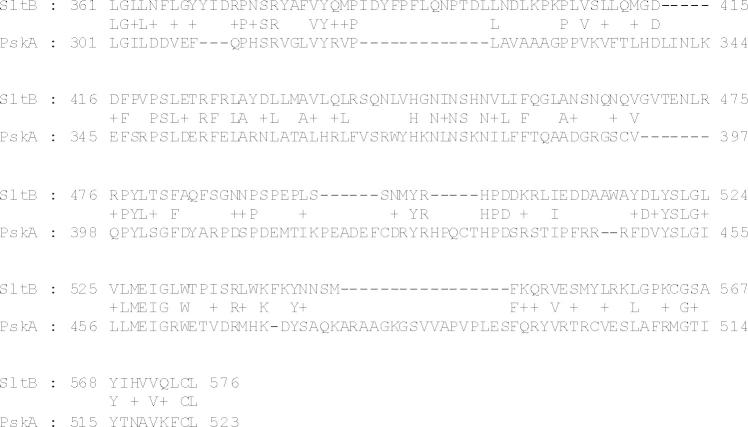
Alignment of the SltB and PskA, AN7985, proteins. The amino acid sequence conservation between SltB and PskA is confined to the pseudokinase domain. Alignment of the SltB fragment between residues 361 and 576, and residues 301-523 of PskA is shown, generated by Blast2p (http://blast.ncbi.nlm.nih.gov/). Identical residues are indicated and + denotes conserved residues using Blosum62 comparison matrix.

Further searches identified, in some fungal genomes, genes encoding proteins having similarity to the N-terminal moiety of SltB but lacking the C-terminal protease-like domain. Genes encoding proteins showing significant similarity only to the C-terminal part of SltB were not found in any analyzed fungal genome. Proteins related to the N-terminal moiety of SltB also lacked important functional residues of Ser/Thr kinases. Thus we predict that these are putative members of pseudo-kinase families. In *A*. *nidulans* we identified AN7985 encoding a putative pseudo-kinase and named it *pskA* (Fig. S5).

### 3.7 Putative pseudo-kinase A, PskA AN7985, is not a component of the Slt pathway

**Supplementary Fig. S6:**
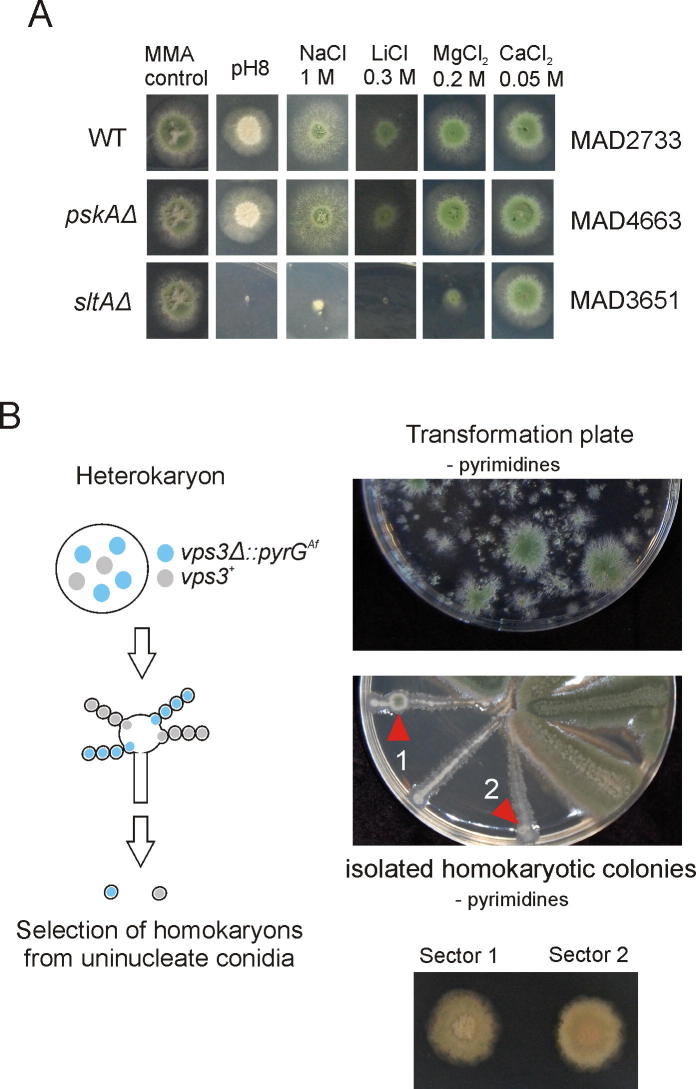
*pskA*, AN7985, does not play a role in the *slt* pathway. A. A null *pskA* strain has a wild-type phenotype under different cation and alkaline pH stress conditions. B. Absence of PskA protein does not suppress the debilitated growth resulting from a null *vps3* allele. However, spontaneous suppressors of *vps3Δ* arise on homokaryon purification plates. Suppressor sectors 1 and 2, indicated with red arrowheads, were purified and further analyzed for the presence of mutations in *sltA* and *sltB* (see Table 2).

PskA is the most similar protein to SltB encoded in *A*. *nidulans* genome. This similarity is restricted to the N-terminal moiety of SltB, between amino acids 361–576 of SltB and 301–523 of PskA, 43% similarity and 26% identity (Fig. S5). As for SltB and SltA, its phylogenetic distribution is limited to the Pezizomycotina subphylum. These aspects prompted us to study its possible role in the Slt pathway. A *pskAΔ* strain (MAD4663) was constructed by gene replacement with the *riboB^Af^* gene in the recipient strain MAD2732 (Table 1), but did not display the cation and alkaline pH sensitivities characteristic of null *slt*^−^ mutants (Fig. S6A). Using the null *pskA* strain MAD5131 we studied suppression of *vps3Δ* and *vps20Δ*. Deletion cassettes for *vps3* and *vps20* were transformed in *pskAΔ* strain MAD5131 and heterokaryotic transformants were selected for complementation of the *pyrG89* auxotrophy (Osmani et al., 2006). Most transformants exhibited the deleterious effect of these null *vps* alleles (a *vps3Δ::pyrG^Af^* deletion experiment is shown Fig. S3B). The poor growth of pyrimidine prototrophic strains after heterokaryon resolution showed that absence of PskA activity does not suppress the poor growth of these *vps* null strains. Sectors with improved growth rates did emerge from some of these *vps3Δ pskAΔ* homokaryons. Two of these sectors were analyzed. In one case the suppressor mutation was *sltB105* and in the other it was *sltA114* (Table 2 and see above). Thus there is no evidence for involvement of PskA in the Slt pathway.

### 3.8 SltA regulates expression levels of sltB

**Fig. 7:**
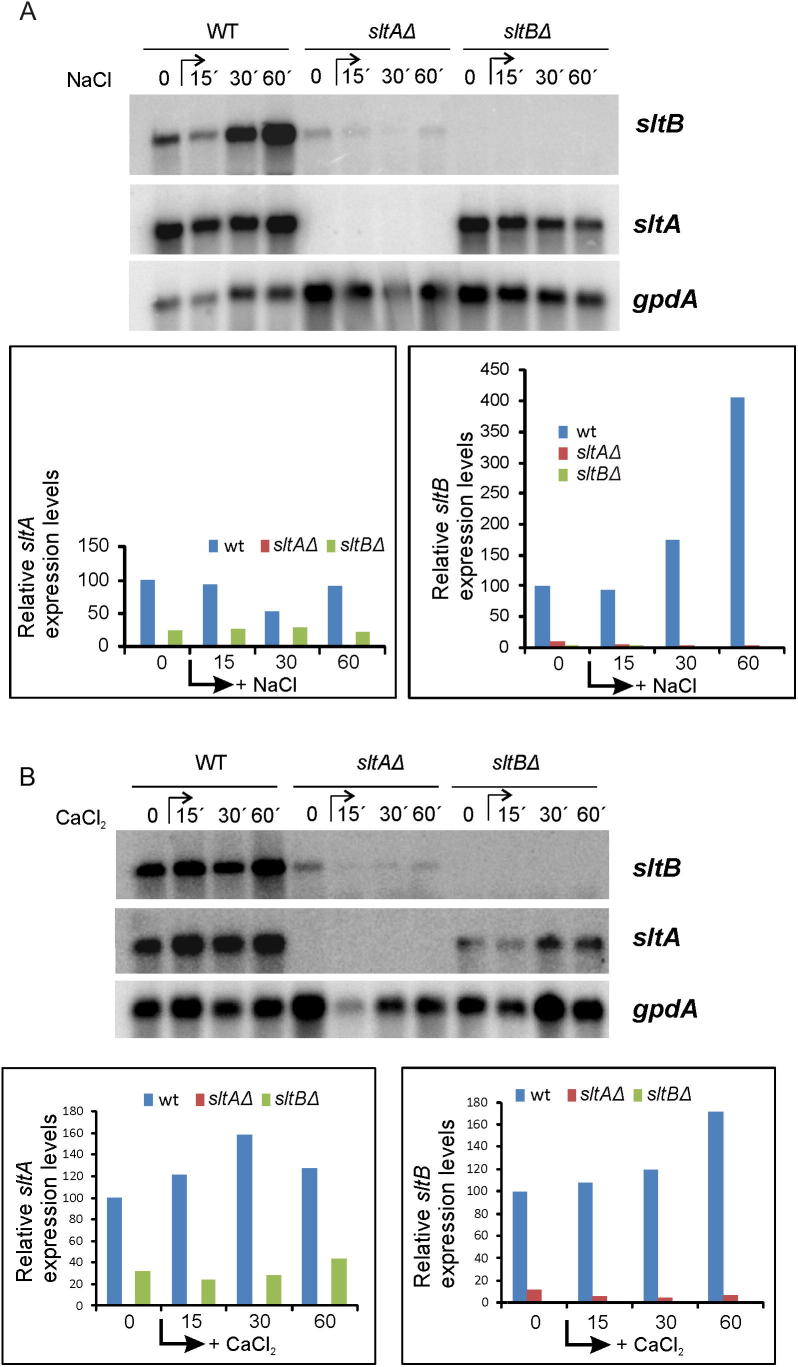
Transcriptional regulation of *sltA* and *sltB*. Transcript levels of *sltA* and *sltB* were analyzed in total RNA samples extracted from mycelia of wild-type MAD2733, null *sltA* MAD3651 and null *sltB* MAD3682 strains grown in the absence or presence of elevated concentrations of cations (1 M NaCl, 10 mM CaCl_2_). Graphs show the quantification of *sltA* and *sltB* radioactive signals in northern blots relative to *gpdA*, which was used as standard. The expression level for each *slt* gene in the wild-type time 0 sample was designated 100%. The expected sizes for mRNA transcripts are: 5046 nt for *sltB*, 3142 nt for *sltA* and 1776 nt for *gpdA*.

We analyzed the expression levels of *sltA* and *sltB* under different growth conditions and in null mutants, using as a control levels of the housekeeping gene *gpdA* encoding glyceraldehyde 3-phosphate dehydrogenase. The ∼3 kb *sltA* and ∼5 kb *sltB* transcripts were readily detectable in total RNA preparations from mycelia of a wild-type strain grown in standard conditions (time 0 in Fig. 7A and B). Addition of 1 M NaCl elevated *sltB* expression 4-fold after 60 min (Fig. 7A). *sltB* transcript levels were also elevated 1.7-fold 60 min after addition of 10 mM CaCl_2_ (Fig. 7B). 1 M NaCl did not have a pronounced effect on *sltA* transcript levels but 10 mM CaCl_2_ elevated them 1.2- to 1.6-fold (Fig. 7B).

However, absence of either SltA or SltB affected transcript levels of the other *slt* gene. In a *sltAΔ* strain, *sltB* transcript levels were reduced to below 10% of those measured in the wild- type. The presence of five consensus SltA binding sites (5′AGGCA3′ (Spielvogel et al., 2008)) within 1.15 kb upstream of the *sltB* coding region (Fig. 2) strongly suggests that SltA directly regulates *sltB* expression. *sltA* mRNA steady-state levels were also reduced 3-fold in a null *sltB* background (Fig. 7). Given that two consensus target sites for SltA are present in the *sltA* promoter (−462 and −538 from the initiating ATG), autogenous regulation of *sltA* expression is possible.

## 4 Discussion

**Fig. 8:**
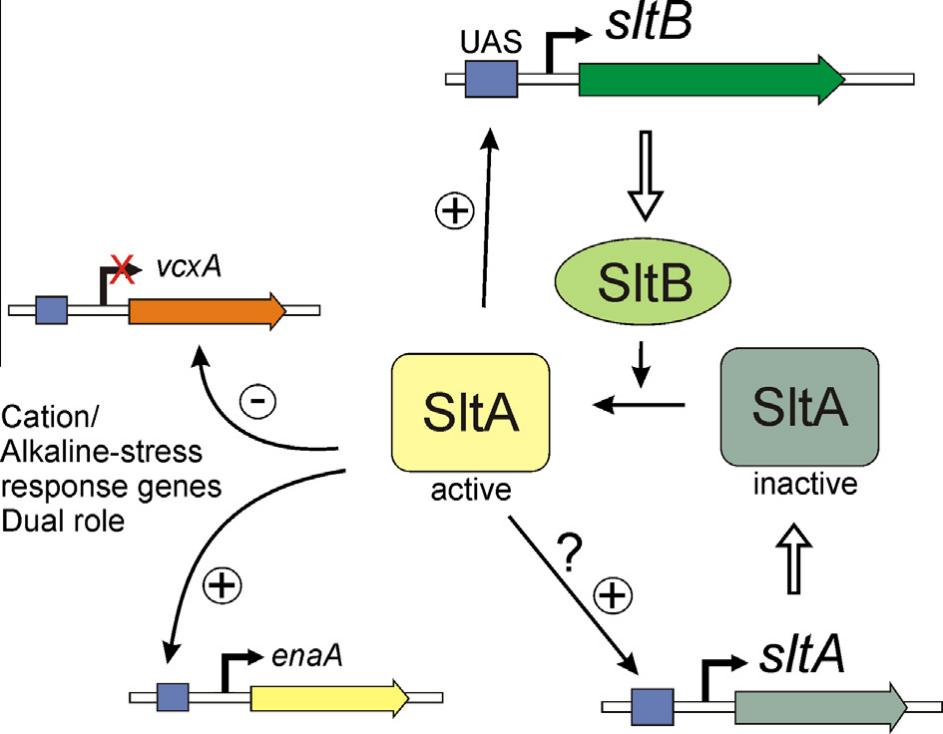
Working model of the *slt* regulatory pathway. The SltA transcription factor alternates between two forms, one active and the other inactive. The SltB protein is a signaling element that mediates conversion of SltA into an active regulator of transcription. Among the genes under the control of SltA are those involved in tolerance to cation stress and alkalinity. SltA has a dual activity (Findon et al., 2010; Spielvogel et al., 2008), acting negatively in the transcription of *vcxA* (a vacuolar cation exchanger coding gene) and acting positively in the expression of *enaA* (a sodium transporter). SltA is also required for the expression of *sltB* and probably is involved in its own transcriptional regulation, in both cases through binding to UAS sequences in the promoter.

In *A*. *nidulans* tolerance to elevated concentrations of monovalent and divalent cations requires the activities of, at least, two zinc-finger transcription factors, CrzA and SltA (Spielvogel et al., 2008). Although elements required for signaling to and activation of CrzA and its homologues are well known (see Hernández-Ortiz and Espeso (2013) and references therein), the SltA signaling pathway was previously unknown. Here we identify and characterize a novel second component of the SltA cation stress responsive pathway. The autocalled locus AN6132 encodes SltB, a 1272 aa protein. Absence of SltB activity results in similar phenotypes to those displayed by the lack of SltA function. Sensitivity to elevated extracellular concentration of alkali metal cations lithium, sodium and potassium and divalent alkaline earth metal cation magnesium is a feature of *sltBΔ* strains. As described for the null *sltA* mutant, calcium is not toxic to *sltBΔ* strains. Not only does calcium improve growth of *sltBΔ* strains but, as with *sltAΔ*, calcium auxotrophy results in double mutants also lacking the HalA kinase. Thus, a clear functional relationship is proposed for SltA and SltB. Predicted functional domains in SltB define this protein as a signaling element for SltA (Fig. 8).

SltB is most probably a bi-functional protein. Searches for conserved domains predicted the presence of a serine/threonine kinase-like domain between coordinates 350 and 550. However the absence of conservation of key residues in functional subdomains of protein kinases strongly suggests that SltB, in fact, contains a pseudokinase domain. Pseudokinases are a growing family of proteins of great interest for signaling and regulation (Boudeau et al., 2006), although some pseudokinases are still able to catalyze phosphoryltransfer reactions (i.e. WNK protein kinases, reviewed in Zeqiraj and van Aalten (2010)). Two major functions have been proposed for pseudokinases, based on the intrinsic protein–protein interaction potential of protein kinases: (1) acting as scaffolds, through recruitment of partners or modulators during the protein phosphorylation process; (2) acting as specific competitors of the actual protein kinases. In most of these cases, the pseudokinase originated through a gene duplication with subsequent loss of functional residues (reviewed in Reiterer et al. (2014) and Zeqiraj and van Aalten (2010)). In the case of the SltB pseudokinase domain there is no kinase encoded in the genome with sufficient similarity to be considered as an “ancestor” or source. In fact, our searches found PskA, AN7985, as the most similar protein to the pseudokinase domain of SltB but it also lacks conserved functional domains for a phosphoryl transfer activity. Again, no identifiable actual kinase partner was found for PskA. This is a first investigation of a possible role for a pseudokinase in *A*. *nidulans*. Putative filamentous fungal kinases, Ffks, have been identified in a systematic analysis of the *A*. *nidulans* kinome; some are suspected to be pseudokinases (De Souza et al., 2013). Neither PskA nor SltB were identified in that investigation, but, interestingly, *pskA* is divergently transcribed to AN7986, denoted *ffkA* in that systematic study. Our data support a model in which SltB must modulate the activity of transcription factor SltA. Further studies will define whether this regulatory activity on SltA is based on a remaining phosphorylation activity or on providing a scaffold to recruit additional elements of this signaling pathway.

The second predicted functional region in SltB is a chymotrypsin-like serine protease domain located in the C-terminal moiety. This prediction is based on extensive searches where a notable similarity to a member of the S65 family of endoproteases was found. The *S*. *cerevisiae* Ssy5p endoprotease is the founding member of this S65 family and the third element of the SPS sensor of extracellular amino acids (Forsberg and Ljungdahl, 2001). Although the similarity of the SltB protease domain to Ssy5p is very low, this comparison has enabled prediction of a possible catalytic triad in the SltB protease domain. Among mutations isolated to date in *sltB*, none modify the putative His925–Asp(1037/1046/1049)–Ser1142 (Fig 5) triad; however, *sltB104*, truncating SltB after residue 1004, demonstrates the important role of this region for SltB activity. The loss of function mutation *sltB56* (G1156R) predicts the presence of additional elements, not conserved with Ssy5p, modulating the activity of SltB near this putative protease domain. In fact there is no evidence that SltB might be a homologue of Ssy5p, and searches did not find identifiable homologues for the other two elements of the SPS sensor or the cognate transcription factor Stp1. In addition, current data do not indicate a role of SltA or SltB in the utilization of amino acids as nitrogen sources. For example, utilization of serine, threonine and proline are not affected (data not shown). However, the functional relationship between Stp1 and Ssy5 can serve as a working model to understand SltB signaling to SltA, since the membrane-associated transcription factor Stp1 is activated through a proteolysis mediated by Ssy5p (Abdel-Sater et al., 2004).

Selection of suppressors of the debilitating growth effect of mutations in ESCRT genes has provided an informative collection of mutations in *sltA* and *sltB* (this work and Calcagno-Pizarelli et al. (2011)). In addition to delineating possible functional domains in both SltA and SltB, the mutations might potentially throw light on the mechanisms mediating intracellular vesicle trafficking in filamentous fungi. The normal distribution and size of vacuoles is disturbed in the absence of SltB, as previously described for null *sltA* mutants (Calcagno-Pizarelli et al., 2011; Findon et al., 2010). Analysis of the available mutants demonstrates that there is not a straightforward relationship between cation stress resistance and suppression of null *vps* alleles. For example, *sltB53*, *sltB56* and *sltB57*, resulting in null, partial loss-of-function and wild-type phenotypes for cation tolerance, respectively, suppress the very poor growth due to a null *vps* allele to similar extents. In contrast to the abundance of mutations truncating SltA, our *sltB* mutations have a high frequency of single amino acid substitutions or deletions. Although a null *sltB* allele is a suppressor of a null *vps* (*i*.*e*. *vps20*) this result suggests that the presence of the various domains in SltB is advantageous to improve viability for *vps* suppression. In fact, those *sltB* mutations affecting the region linking the pseudokinase and protease domains are particularly interesting. Four mutations, *sltB102*, *sltB103*, *sltB106* and *sltB57*, have been selected and the last two, characterized in more detail, have no effect on cation tolerance, indicating that this connecting region may play a more specific role in interacting with the *vps* system. Selection and characterization of additional mutations in this region should be informative. An additional interest of selecting further suppressors of *vps* null alleles is the potential to find new components of the Slt pathway. An attempt to identify new members based on similarities and a pezizomycotina specific distribution has been unsuccessful. The fact that lack of pseudokinase PskA does not affect the cation stress response or suppress *vps*^−^ mutations clearly demonstrates that a classical forward genetics approach will be needed.

Dual positive and negative function was proposed for SltA, with negative regulation of the vacuolar Ca^2+^/H^+^ exchanger gene *vcxA* transcription but positive regulation of sodium ATPase gene *enaA* (Spielvogel et al., 2008). A positive role for SltA in regulation of *nsdD* and *steA* involved in sexual development and sterigmatocystin biosynthesis has also been described (Shantappa et al., 2013). Here we describe a new positive-acting role for SltA in *sltB* transcription (Figs. 7 and 8). The presence of four consensus SltA binding sites in the immediate vicinity of the *sltB* transcription start point supports a model in which SltA directly regulates *sltB* expression. SltA is probably responsible for the elevation of *sltB* expression under sodium stress. Interestingly, absence of SltB also reduces *sltA* transcript levels, indicating a strong functional connection between signaling protein and transcription factor. *sltA* might be subject to autogenous regulation, since consensus target sites for SltA are found in its promoter as tentatively proposed in the scheme in Fig. 8. Thus determining the transcriptional regulation range of SltA and finding additional elements in the Slt pathway might indicate how cation stress is sensed, how signal transduction proceeds toward SltA and how this transcription factor modulates expression of genes under its control. Since SltA and SltB are proteins specifically found in a restricted group of fungi, which includes those of clinical and biotechnological interest, it is of future interest to determine their possible roles in pathways of applied interest, as already determined for the sterigmatocystin biosynthetic pathway (Shantappa et al., 2013), and in pathogenesis.

## Figures and Tables

**Table 1:** Strains used in this work.

Strain	Genotype	Reference
MAD1427	*pyrG89*, *argB2*, *pabaB22*, *nkuAΔ::argB*, *riboB2*	Markina-Iñarrairaegui et al. (2011)
AMC46	*biA1; pyroA4; nkuAΔ::BAR; pyrG89*	Calcagno-Pizarelli et al. (2011)
MAD2732	*argB2*, *pabaB22*, *nkuAΔ::argB*, *riboB2*	Markina-Iñarrairaegui et al. (2011)
MAD2733	*argB2*, *pabaB22*, *nkuAΔ::argB*	Markina-Iñarrairaegui et al. (2011)
ELQ	*adE20*, *suA1adE20*, *mx* (*sltB2*), *yA2*, *pabaA1*, *niiA4*	This study
MAD2461	*sltB2*, *yA2*, *pabaA1*	This study
MAD5440	*pyrG89*, *sltB2*, *biA1*, *halA24*, *pantoB100*	This study
MAD1685	*inoB2*, *sltAΔ::riboB^Af^*	This study^a^
MAD1686	*ΔhalA::pyr-4^Nc^*, *inoB2*	This study^a^
MAD2757	*wA3*, *halAΔ::pyr-4^Nc^*, *inoB2*, *sltAΔ::riboB^Af^*	This study
MAD3114	*biA1*, *yA2*, *pabaA1*, *halA24*, *sltA1*	This study
MAD4048	*pyrG89*, *pabaA1*, *sltA1*	This study
MAD3651	*pyrG89*, *argB2*, *pabaB22*, *nkuAΔ::argB*, *sltA*Δ*::pyrG^Af^*	This study
MAD3919	*pyrG89*, *argB2*, *pabaB22*, *nkuAΔ::argB*, *sltA*Δ*::riboB^Af^; riboB2*	This study
MAD3624	*pyrG89*, *sltBΔ::pyrG^Af^*, *argB2*, *pabaB22*, *nkuAΔ::argB*, *riboB2*	This study
MAD3669	*pyrG89*, *sltBΔ::riboB^Af^*, *argB2*, *pabaB22*, *nkuAΔ::argB*, *riboB2*	This study
MAD3682	*sltBΔ::riboB^Af^*, *argB2*, *pabaB22*, *nkuAΔ::argB*, *riboB2*	This study
MAD3814	*pyrG89*, *sltBΔ::riboB^Af^*, *argB2*, *pabaB22*, *nkuAΔ::argB*, *sltAΔ::pyrG^Af^*, *riboB2*	This study
MAD5441	*sltBΔ::riboB^Af^*, *pabaB22*, *ΔhalA::pyr-4^Nc^*	This study
MAD4663	*pskAΔ::riboB^Af^*, *argB2*, *pabaB22*, *nkuA*Δ*::argB*, *riboB2*,	This study
MAD5131	*pyrG89*, *ΔpskA::riboB^Af^*, *argB2*, *pabaB22*, *nkuAΔ::argB*, *riboB2*	This study
AMC190	*sltB56*, *biA1*, *pyroA4*	This study
AMC196	*sltB53*, *yA2*, *pabaA1*, *pyroA4*	This study
MAD5421	*sltB57*, *pyroA4*, *nkuAΔ::BAR* (*?*), *riboB2*	This study
MAD5423	*inoB2*, *nkuAΔ::BAR* (*?*), *sltA114*, *riboB2*	This study
MAD5425	*sltB106*, *inoB2*, *nkuAΔ::BAR* (*?*)	This study

a Construction of *ΔsltA::riboB^Af^* and *ΔhalA::pyr-4^Nc^* alleles is described in Findon et al. (2010).

**Table 2:** Mutant4 alleles in *sltA* and *sltB* selected in this work.

Allele	Mutation (DNAg)^a^	Derived protein
*sltB53*	ΔTCT after 3376 bp	ΔF1126
*sltB56*	T1434A	Y478^b^
*sltB57*	ΔACA after 1627 bp	ΔN544
*sltB102*	T1702G	Y568D
*sltB103*	A2914C	T972P
*sltB104*	Δ3013–3014 bp	D1005^b^
*sltB105*	G3534A	G1156R
*sltB106*	G2227A	E743K

*sltA100*	G133T	E45^b^
*sltA101*	C1752A	Y549^b^
*sltA112*	ΔT1803-T1808; 1803*ins*GAGGGGG	D566*fs*
*sltA113*	ΔT666-C714	T205*fs*-SSALF
*sltA114*	A1397C	K431T

a Coordinates refer to genomic sequence starting from the start codon.

b Indicates a stop codon. *fs* indicates a frame shift. *ins* indicates an insertion. bp = base pair.

## Appendix A Supplementary material

Supplementary Table S1

Supplementary Fig. S2 The *sltB* gene of *A. nidulans*. The nucleotide and deduced protein sequence is shown for AN6132. 5′ and 3′UTRs are shown. In the 5*’*-UTR the putative transcription start point according to RNAseq data deposited in the AspGD data base is indicated. Also underlined are the consensus SltA binding sites 1 and 2RV (see Fig. 2). The intron sequence, based on type A splicing (Fig. S1), is shown in bold italics.
