# Supplementary material for: A second component of the SltA-dependent cation tolerance pathway in Aspergillus nidulans
Source: Fungal Genet Biol. 2015 Sep;82:116–28. doi: 10.1016/j.fgb.2015.06.002 (PMC4557415; doi:10.1016/j.fgb.2015.06.002)
Supplement: Supplementary Table S1 [file mmc1.docx]

Table S1. Oligonucleotides used in this work.

| Primer | Sequence (5´ → 3´) | Target |
| --- | --- | --- |
| sltB PP1 | GGGAGTATAATAAGGCTGTTGCGGCTT | *A. nidulans* *sltB* promoter |
| sltB PP2 | CTTGGCGACACCGGAGACGACTCG | *A. nidulans* *sltB* promoter |
| sltB SMP1 | CGAGTCGTCTCCGGTGTCGCCAAGACCGGTCGCCTCAAACAATGCTCT | Amplification of *pyrG^Af^*/*riboB^Af^* SM |
| sltB GFP2 | GCACAGTGGACGGGGTAAGGTGAGTCTGAGAGGAGGCACTGATGC | Amplification of *pyrG^Af^*/*riboB^Af^* SM |
| sltB GSP3 | TCACCTTACCCCGTCCACTGTGC | *A. nidulans* *sltB* terminator |
| sltB GSP4 | CGAAAGAAGCTCTACATTGTCCACGAGGAT | *A. nidulans* *sltB* terminator |
| sltBmet | CCAAGATGTCCGTACTCCCGCACCATGG | *A. nidulans sltB* probe |
| sltB477 GSP2 | AGGACGACGGAGGTTTTCAGTCAC | *A. nidulans sltB* probe |
| sltA PP1 | CTGTCTGGCGACCCTGAGAG | *A. nidulans* *sltA* promoter |
| sltA PP2 | GATGGACGGTCCCTAATGTCCG | *A. nidulans* *sltA* promoter |
| sltA SMP1 | CGGACATTAGGGACCGTCCATCACCGGTCGCCTCAAACAATGC | Amplification of *pyrG^Af^*/*riboB^Af^* SM |
| sltA gsp3´ | CCACCAAATGTGACGAGACTGTCTGAGAGGAGGCACTGATGCG | Amplification of *pyrG^Af^*/*riboB^Af^* SM |
| sltA gsp3 | TCTCGTCACATTTGGTGG | *A. nidulans* *sltA* terminator |
| sltA gsp4 | GCTACGGATGCTGACTCC | *A. nidulans* *sltA* terminator |
| sltA met200 | CGGACATTAGGGACCGTCCATCATGGATGCCCAAAGCAC | *A. nidulans* *sltA* probe |
| sltA GSP6 | GAGACCACCAGGGCCGG | *A. nidulans* *sltA* probe |
| AN7985 PP1 | CGGTACAGGTTCTCGGTCGTTAAGA | *A. nidulans* *pskA* promoter |
| AN7985 PP2 | CTCGTATTCGCAAGCGTGAGATATGAG | *A. nidulans* *pskA* promoter |
| AN7985 SMP1 | CTCATATCTCACGCTTGCGAATACGAGACCGGTCGCCTCAAACAATGCTCT | Amplification of *riboB^Af^* SM |
| AN7985 GFP2 | AATCGCAGTACCTATGCTGGGAAGGTCTGTCTGAGAGGAGGCACTGATGCAT | Amplification of *riboB^Af^* SM |
| AN7985 GSP3 | ACCTTCCCAGCATAGGTACTGCGATT | *A. nidulans* *pskA* terminator |
| AN7985 GSP4 | CCTAGGTTTCCAGCTATACCTACCGT | *A. nidulans* *pskA* terminator |
| PRGUP | GAATTGAGCTCGGTACC | 5' flanking region to BamHI site in pRG3 plasmid |
| PRGDW | AAGCTTGCATGCGCGGCC | 3' flanking region to BamHI site in pRG3 plasmid |

* SM: Selectable Marker
