# Supplementary material for: A second component of the SltA-dependent cation tolerance pathway in Aspergillus nidulans
Source: Fungal Genet Biol. 2015 Sep;82:116–28. doi: 10.1016/j.fgb.2015.06.002 (PMC4557415; doi:10.1016/j.fgb.2015.06.002)
Supplement: Supplementary Fig. S2 — The sltB gene of A. nidulans. The nucleotide and deduced protein sequence is shown for AN6132. 5′ and 3′UTRs are shown. In the 5’-UTR the putative transcription start point according to RNAseq data deposited in the AspGD data base is indicated. Also underlined are the consensus SltA binding sites 1 and 2RV (see Fig. 2). The intron sequence, based on type A splicing (Fig. S1), is shown in bold italics. [file mmc2.docx]

**Figure S2**

-300 tcaaagtgg**ttgcctg**ccacgtgattctcccgatctgtggcttggcgatgaggctttgct

-240 tcactcgccactccttattggt**taggcag**cacaattccctcccacagccaaggtattctt

TSP

|

-180 attgcaacctgctcctctcctgcagccttctcctaactttctcgtctatcaacgtcgtgc

-120 tgttagtgcttcaatatttctggctttatccgctcgctcaacgacagcgctcgcctctga

-60 aatgttctgagaccaacccccgcccccgcatccgcccgagtcgtctccggtgtcgccaag

1 atgtccgtactcccgcaccatgggggttcatacctccagcggcgcctcacgaagatgtat

M S V L P H H G G S Y L Q R R L T K M Y 20

61 accgacaccaagacatcttgtgagagtgtcactaccgcctccaacgccgctgatgacccc

T D T K T S C E S V T T A S N A A D D P 40

121 gagcttgtttccatccaccgtaatttcaagacacagagggataggcttctggcgtggggt

E L V S I H R N F K T Q R D R L L A W G 60

181 ttggactggagcgacgcgagcgccgctcagccgaacgacatcgacgaatcgctgacgcaa

L D W S D A S A A Q P N D I D E S L T Q 80

241 gctggttttagtgatgtggtggaaagcgtaatgtcgtcgattcaagatatactcagcgag

A G F S D V V E S V M S S I Q D I L S E 100

301 gcggaacggctacagaataagggcgcctcagatctacctccgaagggaggtaaagcggag

A E R L Q N K G A S D L P P K G G K A E 120

361 tctcccgcggctgatggactcggcggtttctcgaacaagaccaactggaccagcgctgag

S P A A D G L G G F S N K T N W T S A E 140

421 atcgctcgttccaagaccctgctcactgagttgacggcatgcatcgacgtcctttacgac

I A R S K T L L T E L T A C I D V L Y D 160

481 ctttctggctcgcgccgcgatatgaccgccaatgcacagccttcgggtaagagtcggacc

L S G S R R D M T A N A Q P S G K S R T 180

541 cggtcttatccctcaccccaggtcggcagtcctggcggtgatctgggccgcggaaacctt

R S Y P S P Q V G S P G G D L G R G N L 200

601 cagaactccaaatcatctcatggtgcctcctttgatagtacgcaaaggcatcagagtccc

Q N S K S S H G A S F D S T Q R H Q S P 220

661 cagtcaccgaagtccaaggaggcgtttgaatactcttctttcaccaatctccatgccatc

Q S P K S K E A F E Y S S F T N L H A I 240

721 acccagtcacctgtcttcgaacaaccaaagttcaataaagctttcagtaaagcctccgtc

T Q S P V F E Q P K F N K A F S K A S V 260

781 gaaccaaagaggtatctaatcgaccgttcagcattacagctaaccggtgcatcccacgac

E P K R Y L I D R S A L Q L T G A S H D 280

841 aataatccacctccatatgagatggtggccgcttccacaaactctcgagtccttggccgc

N N P P P Y E M V A A S T N S R V L G R 300

901 atgaaaacatcagctctacctttccgccacaacttaaaggatgctactgttagcatcctc

M K T S A L P F R H N L K D A T V S I L 320

961 gttgaatacaccccaatggtacttgactcgtcgtctgaacctccctatcccggagatgca

V E Y T P M V L D S S S E P P Y P G D A 340

1021 cggcttgataatgtgcatcagacacttgatcagctggtccagaatgcgcgggtctcccac

R L D N V H Q T L D Q L V Q N A R V S H 360

1081 cttggactactgaacttccttgggtactacattgatcgacccaattcccgttacgctttc

L G L L N F L G Y Y I D R P N S R Y A F 380

1141 gtctatcaaatgccaatcgactatttcccgttcctccagaaccctaccgaccttctaaat

V Y Q M P I D Y F P F L Q N P T D L L N 400

1201 gatctgaagcctaagcccttggtttcgcttctccaaatgggagacgactttccggtaccg

D L K P K P L V S L L Q M G D D F P V P 420

1261 agcctggagactaggttccgcttagcttatgacctccttatggctgtattgcagctcaga

S L E T R F R L A Y D L L M A V L Q L R 440

1321 agtcagaatctcgttcacggaaatatcaacagccataatgttctcatcttccaaggtctg

S Q N L V H G N I N S H N V L I F Q G L 460

1381 gcaaactcaaatcaaaaccaagttggagtgactgaaaacctccgtcgtccttatcttacc

A N S N Q N Q V G V T E N L R R P Y L T 480

1441 tccttcgctcagttctccgggaacaacccgtctcccgagccgctttcttcgaatatgtac

S F A Q F S G N N P S P E P L S S N M Y 500

1501 cgccatcccgacgataagaggctgatcgaagatgatgcagcctgggcatacgatctctac

R H P D D K R L I E D D A A W A Y D L Y 520

1561 tctctcggcttggtcctgatggagattggcctttggacgccgatcagtcgcctgtggaag

S L G L V L M E I G L W T P I S R L W K 540

1621 ttcaaatacaacaactctatgttcaagcaaagggtggagagtatgtatctgaggaagcta

F K Y N N S M F K Q R V E S M Y L R K L 560

1681 ggccccaagtgcggtagtgcatacattcatgtggttcaattatgtctcgatgcaccaaat

G P K C G S A Y I H V V Q L C L D A P N 580

1741 ttccacctttcgacccagccatttgatgacctcaacttgagaatacctcagaccttccac

F H L S T Q P F D D L N L R I P Q T F H 600

1801 tacccggtcttggatctttcggcacccgagagcacttttgctttctccatgaatttcgtt

Y P V L D L S A P E S T F A F S M N F V 620

1861 tacaccttgtgtaaaataacttggtcgtgctgcaggattgacattttttctgctccagca

Y T L C K I T W S C C R I D I F S A P A 640

1921 gctgaagagctggacgattgtcttcctctagcgcttgtccctggttccgaagcagatgct

A E E L D D C L P L A L V P G S E A D A 660

1981 gcaaagcaagctgcgcgagaatacaagggacctgaacaagtaacccgcttctcgcaacca

A K Q A A R E Y K G P E Q V T R F S Q P 680

2041 gtggctgtggctcttacccctgagaccaaattaatgaagatcgggcttgaagaaaaaagg

V A V A L T P E T K L M K I G L E E K R 700

2101 ctgaggaagcgcacattcaagaaacttacaacggtcgaaattcctcaagaacacctcgat

L R K R T F K K L T T V E I P Q E H L D 720

2161 gagtggaacttccgcatgctgcccaaattgaggaagctgctgcagaaggtattgaaggat

E W N F R M L P K L R K L L Q K V L K D 740

2221 tcgtctgaatcatgcggtgtcactctcatgatgacgggcgacggtttggaaaatgcgaag

S S E S C G V T L M M T G D G L E N A K 760

2281 acaacaatttgtgtgacttgtgctagcgttaagaaggttagggcggcgctgaagaagtac

T T I C V T C A S V K K V R A A L K K Y 780

2341 ttccctcttgatgacaaagaagactgggacttgcttgtgcttcgcggagatattgagcga

F P L D D K E D W D L L V L R G D I E R 800

2401 tccaaagtcccgcgcaagaagcgccgccgccctgcaaagactggacctcttagcactatc

S K V P R K K R R R P A K T G P L S T I 820

2461 gaagcccctcctcctccgcaggaccctaacccctgttttcagccgaggcctctctgcggt

E A P P P P Q D P N P C F Q P R P L C G 840

2521 gcatccatgggcgccttcatgaatgaagaacatctccctcctgtgtcgtacggtggtgcc

A S M G A F M N E E H L P P V S Y G G A 860

2581 atcctagttgatggtgttccctacggtatgacagtacatcacatgctcgaggctcccagc

I L V D G V P Y G M T V H H M L E A P S 880

2641 gatcaagaggatgcggatgatatacatgaagctttggaggatgctctgcttcggtcggcc

D Q E D A D D I H E A L E D A L L R S A 900

2701 ggaaactggactcgcgatctggcttcccagaattcacagctcatgtcagcttggaatgat

G N W T R D L A S Q N S Q L M S A W N D 920

2761 gactcctctgcacatagtctcgaatttgaagtctccgatgacgaggatggcgacgactac

D S S A H S L E F E V S D D E D G D D Y 940

2821 tatgatgactttgcgctctctgaaggatattcatctgacgagggcgacgacgactttgct

Y D D F A L S E G Y S S D E G D D D F A 960

2881 tacgatgacgatgacagagcgtcgatcggagacacagctggcatcgaacctggagaagag

Y D D D D R A S I G D T A G I E P G E E 980

2941 cctcccgtgtttgtaacccagcctgccattgatgacgttcgcgaagatttctttccaagc

P P V F V T Q P A I D D V R E D F F P S 1000

3001 cccgaggacagagatgacgagcatctcgcttcccacactttcggatacgtttatgcttcc

P E D R D D E H L A S H T F G Y V Y A S 1020

3061 tcaggcgttcgacgctggacacgacagggcatcaagcatgaaatcgactgggctttgatt

S G V R R W T R Q G I K H E I D W A L I 1040

3121 aagatcaaccaggatcgcttagacgcgaggaatattgtctacgacaagccgtcctactct

K I N Q D R L D A R N I V Y D K P S Y S 1060

3181 ttagtggcctcagcgagacggagaccgggtgaaccgatgcccctacaacaacggaacccc

L V A S A R R R P G E P M P L Q Q R N P 1080

3241 atcctcttgaatgacatagcacggtttgaggagcttggtggcctcaatgttcactgctgt

I L L N D I A R F E E L G G L N V H C C 1100

3301 ggccgaactagcgggttgcaaactggacagatttcgaaggcgttgacgatggtgaagcta

G R T S G L Q T G Q I S K A L T M V K L 1120

3361 catggtcggcacaccttctccacgagctttggggtggatgggaacttcggag***gtgagtga***

H G R H T F S T S F G V D G N F G 1137

**3421 *gtgttgcttgccagcattctcgttaactttctcccttctcatactaacctggctctttag***

3481 ttcccggtgattctggcgcttgggtctttgaaaaatccactggccgcgtttgtggagctg

V P G D S G A W V F E K S T G R V C G A 1157

3541 tcctggcatggtccgaggcacttcgcatcgcctatatcgctccaatggaggtcctccttg

V L A W S E A L R I A Y I A P M E V L L 1177

3601 aagacatcgctcgcaccctacacgcgacccatgtcacacttccagatggccctaacgagt

E D I A R T L H A T H V T L P D G P N E 1197

3661 ctgtgacctttcctatgcagcacccgtccgttcccccaaatcctagatacctcggcccgc

S V T F P M Q H P S V P P N P R Y L G P 1217

3721 ctccattgccagatcaacttcatgttgatctcaaccgacaactgcatcttgatgatcaga

P P L P D Q L H V D L N R Q L H L D D Q 1237

3781 gcgttggtgcagtcaacaccagtcgtgggctcagagagccataccgaaatctaccgccta

S V G A V N T S R G L R E P Y R N L P P 1257

3841 ttctcaccccagggccgggtcgcagtctggaaagacagcttgcttaatcaccttaccccg

I L T P G P G R S L E R Q L A - 1272

tccactgtgcttaggtacctcacttgtttctcaagtatataccccgtcactgtatgactt

tattttcctaccccacgacgaccctttctcatgaccccgttgcttttgatataatacccc

ctgcgcaaacttgatatgagcactttgacttataaataaataatccttttgaaacgcaat

cgctttcaaagtctagatgagcatgtcaatggccgaacgtataatacagtgcgattagat

gtccaccgtcgcaacaaatatcggaacataccgggggtgtgcaccaaaaaagtcttcgcc

gcactagtgacatttgatgtcaagtcctgtaatacaaatacaggtcattagacaagtagc

tttaacttaagttgagatatagatctcccaaagcaaacaacataacttttcctcactttc

gaggcttcccccagatggctatcacaaatcaaacatttgtttatcgttctaagcgctttt

tcttccacatttgcctctctgcttctacttctgcctccgaaccgtgtgcgccctttcctg

tctgcgatatctacatgccagcgccttatccaaacgagcttcgagttcaaatcctttcct

actaggttggatctagtcaacagatatagccaagatgcttcaagtcaacatttgaacaac

acgagacatattccagaagggtcaagatcgtggctacaatcctgctcaatgcagagggtt

aaacttgaatatgtggaggatggcaagtgactgggcgtccaaaggagactcctaaagata

cagaaccataaggcatatacaggagaattatacgacataatggggacaatatgtacagat

ggccgtgagcgctatcttgcttgaaactggatgggcggacagatgaagggccagctttct

gcgcgtgaacacctattcccagagccctgaaaacctctggatagcttcatttctgctagt

tttggcgtttttattgtattctct
